# Aqueous Processed Biopolymer Interfaces for Single-Cell Microarrays

**DOI:** 10.1021/acsbiomaterials.9b01871

**Published:** 2020-04-06

**Authors:** Vittorio Ferrara, Giovanni Zito, Giuseppe Arrabito, Sebastiano Cataldo, Michelangelo Scopelliti, Carla Giordano, Valeria Vetri, Bruno Pignataro

**Affiliations:** † Dipartimento di Scienze Chimiche, Università di Catania, Viale A. Doria 6, 95125 Catania, Italy; ‡ Dipartimento di Promozione della Salute, Materno-Infantile, Medicina Interna e Specialistica di Eccellenza “G. D’Alessandro” (ProMISE), Sezione di Malattie Endocrine, del Ricambio e della Nutrizione, Università di Palermo, Piazza delle Cliniche 2, 90127 Palermo, Sicilia, Italy; § Dipartimento di Fisica e Chimica−Emilio Segrè, Università di Palermo, Viale delle Scienze, 90128 Palermo, Italy

**Keywords:** inkjet printing, biopolymer, single-cell, microarray, biointerface

## Abstract

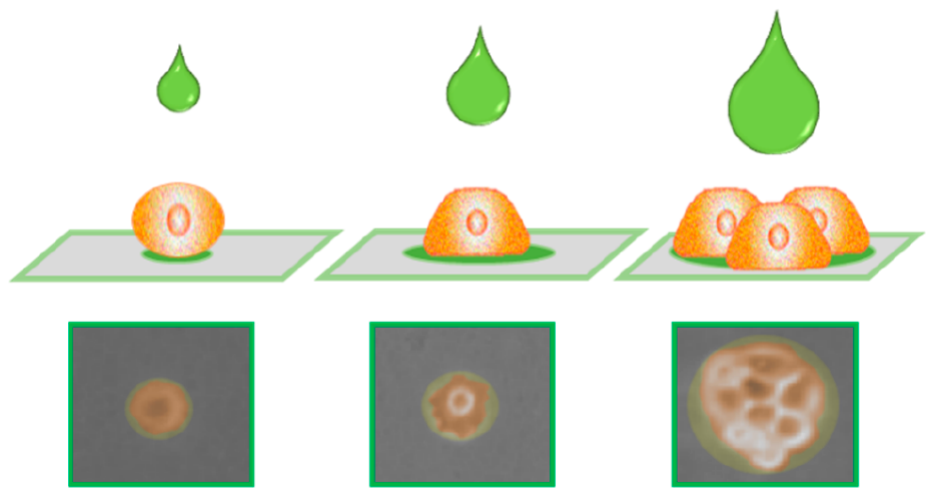

Single-cell microarrays are emerging tools to unravel intrinsic diversity within complex cell populations, opening up new approaches for the in-depth understanding of highly relevant diseases. However, most of the current methods for their fabrication are based on cumbersome patterning approaches, employing organic solvents and/or expensive materials. Here, we demonstrate an unprecedented green-chemistry strategy to produce single-cell capture biochips onto glass surfaces by all-aqueous inkjet printing. At first, a chitosan film is easily inkjet printed and immobilized onto hydroxyl-rich glass surfaces by electrostatic immobilization. In turn, poly(ethylene glycol) diglycidyl ether is grafted on the chitosan film to expose reactive epoxy groups and induce antifouling properties. Subsequently, microscale collagen spots are printed onto the above surface to define the attachment area for single adherent human cancer cells harvesting with high yield. The reported inkjet printing approach enables one to modulate the collagen area available for cell attachment in order to control the number of captured cells per spot, from single-cells up to double- and multiple-cell arrays. Proof-of-principle of the approach includes pharmacological treatment of single-cells by the model drug doxorubicin. The herein presented strategy for single-cell array fabrication can constitute a first step toward an innovative and environmentally friendly generation of aqueous-based inkjet-printed cellular devices.

## 1 Introduction

The investigation of cellular systems at the single-cell level, i.e., single-cell biology, permits one to shed light on their relevant biochemical and biophysical processes at an unprecedented level of detail.^1,2^ Typically, populations of cells are investigated in standard cell cultures conditions, so the resulting extracted biological information does often consist of a broad average output from a cell population. However, it is widely accepted that each cell in tissues and organs can play a unique biological role, potentially showing significant differences when triggered with identical stimuli.^3^ For instance, in the case of the biology of cancer, cell-to-cell heterogeneity triggers tumor progression and growth as well as emergence of drug resistance.^4^ Therefore, single-cell investigation approaches provide the ultimate level of resolution in our quest to capture relevant heterogeneities, constituting versatile tools for disease biomarkers identification,^3^ drug discovery,^5^ intracellular fluorescence-based molecular tracking,^6^ and stochastic gene expression.^7^ Furthermore, single-cells platforms offer a way to manipulate cells at the intracellular level^8^ and to study rare or hard-to-separate mixtures of cell types^1^ with a high-throughput capability.

The emerging field of single-cell biology has been fueled by the development of innovative physicochemical strategies allowing for cellular harvesting and investigation by material-functionalized solid–liquid interfaces^9−11^ that can be ultimately leveraged for the fabrication of cell-capture microarray devices. For example, nonadherent single-cells microarrays were obtained by physically trapping individual cells in polymeric microchambers that can accommodate only one individual cell per well.^12,13^ Also, single cells have been caught by microfluidic approaches onto functionalized materials, whose surfaces have been chemically tailored, in order to introduce cell-adhesive properties and biological selectivity.^14,15^ An even further control of the spatial arrangement and material composition of the cell-capture features becomes crucial allowing for the development of functional single-cell biology platforms.

In this regard, printing techniques allow for the direct fabrication of a large variety of biomolecular structures at micro- or nanoscale resolution and have, in fact, been extensively pursued to obtain adherent single-cells microarrays.^16,17^ For instance, microcontact printing and scanning probe-based methods, such as dip pen nanolithography and polymer pen lithography, allow for the direct fabrication of cell-adhesion micropatterns to control the attachment area, inducing stimuli on single cells down to the subcellular scale (<10 μm).^18−21^ Nevertheless, the reported approaches necessitate high-cost instrumentation and potentially harmful materials processed under vacuum or anhydrous conditions.^22^

Among all the printing approaches, inkjet printing is one the most convenient tools for the modification of cell adhesive solid supports^23^ by dispensing picoliter scale droplets at high-throughput capability (10 spots/s), microscale resolution,^24^ and promising multiplexing potentialities.^25^ Generally, inkjet printing constitutes a very convenient alternative for thin films deposition with respect to conventional approaches, such as spin coating, drop casting, and soaking. In fact, inkjet printing is featured with ambient operative conditions, low material consumption (each droplet is typically on the picoliter-scale, and all the printed materials are used for film fabrication), and high flexibility over the deposition process (in terms of printing speed and pattern design and thickness).^25^

In fact, the inkjet process is suitable for printing an extraordinary variety of inks containing polymers, biopolymers, and biomolecules^26^ under optimized conditions by tuning the ink rheological properties^27^ and the droplet jetting conditions to preserve biomolecules functionality^28^ and at lower costs in comparison to scanning probe-based methods. In the context of cellular biochips, inkjet printing has been exploited as a suitable approach for patterning cells through two different strategies: (1) direct patterning of living cells onto a solid substrate and (2) printing bioadhesive materials in an array format onto a cell-repulsive support to harvest cells on the pattern. The former approach has demonstrated the possibility to dispensing cells with high spatial control.^29,30^ However, the latter strategy is often advantageous with respect to the direct printing of cells. This is due to the fact that the printing process can affect the viability of cells due to the shear stresses that occur during the droplet formation and impact that can result in cell lysis and finally death.^31^ Therefore, the inkjet technology has been mainly used to indirectly pattern adherent cells by fabricating bioadhesive molecules micropatterns at resolution of typically hundreds of microns, ultimately resulting in cell consortia with a defined spatial arrangement.^32,33^ In fact, the commonly employed nozzle size, which typically span in the 20–50 μm regime, eject drops with volumes in the 1–100 pL range, which generally spread on solid supports defining microscale patterns. An interesting example of an inkjet-printed array suitable for cell harvesting at the lower dimensional scale (∼10 μm) has been shown patterning fibronectin spots on a solid hydrophobic support.^34^ This approach has demonstrated the feasibility of trapping individual cells on an ordered inkjetted pattern with high positional control, resulting in a single-cell array. However, the involvement of organic solvents and hazardous compounds, especially organo-silanes for surface chemistry reactions on solid supports, are still present in most of the strategies for biochip fabrication.^18,34,35^ In fact, most of the strategies for biochips fabrication rely on the use of aqueous inks containing cell-adhesive polymers, but often the surface chemistry of the support is modified in preprinting steps by toxic chemicals in organic solvents, as in the case of the glass silanization, a common approach for chemical modification of glassy substrates.^34^ To our knowledge, there are no examples reporting on single-cells arrays produced by inkjet printing through an all-aqueous and green strategy. Indeed, the use of aqueous based inks is of utmost relevance for biomedical application of cellular chips, in order to reduce the presence of potential cytotoxic compounds in the culture system and lower the production of harmful waste during the materials processing and biochip fabrication. The absence of hazardous chemicals is also a fundamental aspect toward the scale-up process, since it reduces the environmental impact of innovative devices production.

In this work, inkjet printed collagen micropatterns able to harvest and arrange single cells in an array fashion with high positional control are presented. To do that, we exploited the feasibility to generate sub-picoliter droplets through micrometric nozzles by inkjet printing, which has only recently been demonstrated in the context of emulsion preparation^36,37^ or by employing more complex setups.^38,39^ The developed printing methodology allows one to accurately modulate the ejected drop volume with the aim of fabricating microarrays with tailored spot dimensions in order to control the number of adherent cells, ranging from single-cell arrays to patterns of cells consortia. Moreover, the herein presented approach combines fabrication at low-cost and scalable conditions provided by inkjet printing by means of environmentally sustainable green materials fully processed in aqueous environments and low temperatures. All the inks are formulated by using water as a solvent and contain glycerol at 20% v/v in order to tune the rheological properties by achieving ideal printability. In fact, glycerol interferes with intermolecular interactions between polymer chains by the formation of hydrogen bonds,^40^ finally reducing clogging at the nozzles. A novel green-chemistry aqueous-processed biointerface is shown, suitable for the fabrication of a single-cell biochip platform, on which efficient drug uptake was also demonstrated.

## 2 Experimental Section

### 2.1 Chemicals

Chitosan (medium molecular weight, 75–85% N-deacetylated), poly(ethylene glycol)diglycidyl ether (EPEG, average *M*_n_ 500), collagen (type I from calf skin, 0.1% solution in 0.1 M acetic acid, aseptically processed), acetic acid (HAc), bovine serum albumin-fluorescein isothiocyanate (FITC-BSA) conjugate and glycerol were all purchased by Sigma-Aldrich. Doxorubicin hydrochloride (Dox) was purchased from Selleckchem (Houston, TX). Sypro Orange and Alexa Fluor 647 succinimidyl ester dyes were purchased by ThermoFisher Scientific. The working solutions and medium for cell culture were purchased by Euroclone, Milan, Italy. All the chemicals were used as received without any further purification. Ultrapure water (direct Q-UV filtration system, 18.2 MΩ) was utilized for all the solutions preparation and washing steps.

### 2.2 Biochip Fabrication

Glass coverslips (Corning, borosilicate glass, thickness 0.13–0.16 μm) were cleaned by two sonication steps of 5 min each in Hellmanex detergent 2% v/v and in Millipore water. Then, they were treated twice by a UV–O_3_ cleaner (Bioforce Nanosciences) for 20 min, rinsing with ultrapure water, and drying with N_2_ steam flow after each treatment. Chitosan ink (0.1% w/v, glycerol 20% v/v, HAc 1% v/v) was dispensed on the as-cleaned glass substrates by using a Dimatix Materials Printer (DMP-2800, Fujifilm, Figures 4 and 5). The chitosan ink was loaded in user-fillable piezo-based inkjet print cartridges, with 16 nozzles, with 254 μm spacing and 21.5 μm in diameter, to print droplets of ∼10 pL volume. The droplets were printed at 40 V by a conventional double pulse waveform (i.e., the voltage vs the time signal given as an input to the piezoelectric actuator)^41^ and spaced 50 μm in order to have coalescence at the interface and achieve an ink film on the glass. All the volatile components of the ink were evaporated by a thermal curing step (from 50 to 100 °C in 20 min and 1 h at 100 °C) obtaining a chitosan thin coating. Pure EPEG (∼1 μL/mm^2^) was drop-casted onto the chitosan coated glasses leaving the grafting reaction to occur overnight at room temperature, then rinsed with ultrapure water. Finally, the collagen ink (0.08% w/v, glycerol 20% v/v, HAc 0.1 M) was loaded in piezo-based cartridges, with 16 nozzles 254 μm spaced and 10.5 μm in diameter, to print droplets of ∼1 pL volume. The collagen ink was printed onto the chitosan/EPEG surface at 30 V by a customized waveform with a single pulse duration of 5 μs, in order to obtain a 150 μm spaced droplets array. All the volatile components of the ink were evaporated by a thermal curing from 50 to 90 °C in 20 and 5 min at 90 °C. A scheme of the printing pattern is reported in Figure S1 (Supporting Information).

### 2.3 Biochip Characterization

#### X-ray Photoelectron Spectroscopy (XPS)

Spectra were recorded with a PHI 5000 VersaProbe II scanning XPS microprobe using monochromatic Al Kα radiation (*h*ν = 1486.6 eV) from an X-ray source operating at 200 μm spot size, 50 W power, and 15 kV acceleration voltage. The takeoff angle was set at 45°.

#### Atomic Force Microscopy (AFM)

Measurements were performed by using a Dimension Icon Instrument (Bruker, Germany). All images were acquired by using RTESP type silicon probes (Bruker, Germany) and collecting 512 × 512 points per image by maintaining a scan rate of about 1 line/s or lesser. As for the surface roughness, the root-mean-square (*R*_q_) and arithmetic (*R*_a_) roughness values were calculated from 5 μm × 5 μm images.

#### Confocal Laser Scanning Microscopy (CLSM)

Fluorescence measurements were acquired by means of an Olympus FluoView1200 confocal laser scanning microscope (Olympus, Tokyo, Japan) using an UPLSAPO 40X oil objective. Doxorubicin fluorescence signal was acquired under excitation at 488 nm in the 560–610 nm range., Sypro Orange and FITC-BSA signals were acquired under excitation at 488 nm in the 515–600 nm range, while Alexa Fluor 647 signals were acquired under excitation at 635 nm in the 650–700 nm range.

#### Cell Culture

The human nonsmall-cell lung cancer (NSCLC) cell line H1975 was provided by Prof. Rolfo (University of Antwerp) and selected for single-cell cultures. H1975 cells were cultured as a monolayer in RPMI 1640 medium supplemented with 10% fetal bovine serum (FBS) and 100 U/mL of penicillin and 100 μg/mL streptomycin. As cells reached the 90% of confluence, they were treated with trypsin/EDTA and centrifugated at 1300 rpm for 5 min. The medium was removed and the cell pellet was resuspended in fresh RPMI medium, according to the optimized cell dilution (1:50, after different dilution tries). Before cell seeding on the printed platform, the printed area on the biochips was framed with an adhesive HybriWell, which surrounded the area containing the collagen array. It allowed to reduce the total amount of working solutions at the microliter-scale. Then, the system was sterilized for 30 min under UV light and equilibrated with PBS 1× for 2 min before cell seeding. Following, the cells can be seeded on collagen array and incubated for 1 h at 37 °C in a humid incubator with 5% CO_2_. It was verified that the UV treated chitosan-*g*-EPEG surfaces did not allow for H1975 cell adhesion. At the end of the incubation on the microarrayed surfaces, every plate was washed several times with PBS 1× in order to remove the nonattached cells. Then, the cell attachment to the collagen spots was evaluated on an inverted optical microscope (Zeiss Axio LabA1). The resulting pattern of cells was at this point cultured in 200 μL of complete RPMI medium for all the further experimental procedures. Analogous conventional cell cultures were carried out in Labtek II (nunk) 8-well chamber slides suitable for CLSM imaging as a reference system. Conventional cultures and single-cell array were treated with Dox 100 μM for 1 h before imaging.

## 3 Results and Discussion

**Figure 1:**
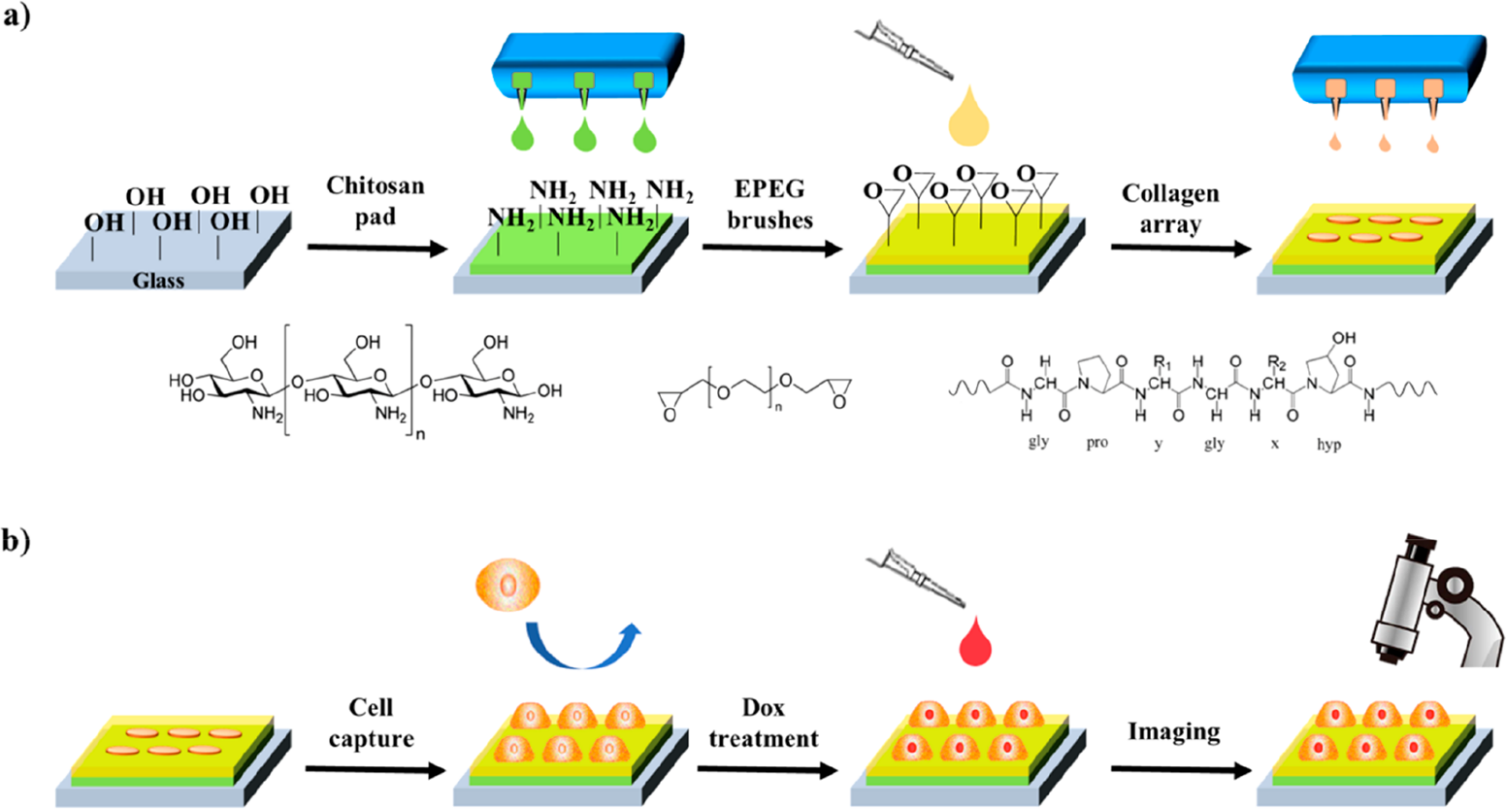
Schematic illustration of single-cell array fabrication and application: (a) layer-by-layer printing steps from chitosan coating deposition on glass to EPEG grafting and collagen microarrays patterning and (b) single human cancer cells capture on the printed biointerface, followed by cell treatment with doxorubicin and analysis by confocal microscopy at the single-cell level.

The procedure for the fabrication of the herein reported green all-polymeric single-cell biochip consists of a multilayer deposition method, in which an inkjet-printed array of collagen spots on a cell-repellent background enabled the capture individual cells with high efficiency. In Figure 1, a sketch of each production step of the biochip and its application at the cellular biointerface is illustrated. The design of the chip preparation was rationalized to obtain high precision order in collagen spots array on a nonfluorescent, biocompatible, nonreactive substrate suitable for confocal imaging, AFM analyses, and common spectroscopic investigations. The chip was developed in planar format on a glass support, whose surface chemistry was first modified after the cleaning procedure by printing a chitosan thin film. Chitosan is a nonfluorescent amino-rich polysaccharide, widely considered a well-film forming biopolymer.^42^ The deposition of a chitosan coating on glass is aimed to obtain surface-exposed primary amino groups at the interface to covalently graft brushes of EPEG drop casted in the following step. The epoxy groups were exploited for the chemisorption of the printed collagen in an array fashion permitting one to define the single cells attachment area. Indeed, the sterilization process of the printed platform by UV was also useful for the deactivation of the epoxy groups of the EPEG structure^43,44^ that otherwise would favor cell adhesion on the collagen-free regions.^45^ The result of this step is a hydroxy-terminated PEG interface, which behaves as a nonfouling background, resisting nonspecific protein adsorption and cell adhesion onto the areas not covered by collagen spots. As well-known, collagen is one of the main components of the extracellular matrix and thereby represents a suitable material for cell attachment and colonization.^46^ The obtained device is proved to efficiently drive single cell patterning on the solid support. Indeed, it permits optical imaging of cell arrangement and morphology.

The polymeric layers were all deposited from aqueous inks following a green approach and characterized at each deposition step by surface analysis techniques, including atomic force microscopy (AFM) and X-ray photoelectron spectroscopy (XPS). Moreover, proof-of-principle of the biochip functionality was evaluated by intracellular delivery of doxorubicin (Dox), a model drug for human cancer therapy.^47^ Drug uptake by single cells and subsequent nuclear localization was proved by fluorescence confocal laser scanning microscopy (CLSM).

### 3.1 Chitosan Film Printing

**Figure 2:**
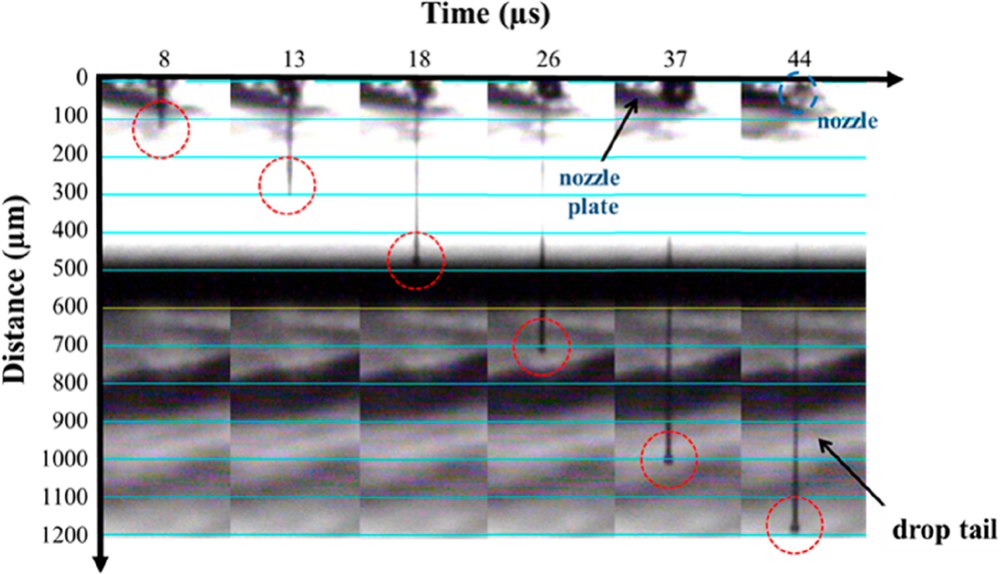
Stroboscopic image of 10 pL (nominal volume) chitosan ink droplet pinching-off at the nozzle at 40 V and room temperature. The drop elongates as a function of time while modifying the drop front (red circles). The arrow in the last step indicates the drop tail (about 800 μm length).

In principle, the fabrication of chitosan films can be carried out by different solution dispensing approaches.^48,49^ For instance, spin coating is a very well-known method,^50^ given its simplicity and the high control on the film thickness. Differently, the inkjet printing approach has received a large interest in the literature,^51,52^ due to its superior capability in the fabrication of patterned coatings with bespoke geometries, along with high control on thickness, roughness, and in scaling-up the devices manufacturing processes as well. Accordingly, the chitosan ink was printed by inkjet printing on glass in order to form an amino-rich polymeric coating, suitable for the further chemical functionalization of the substrate surface. Chitosan, in its protonated form (p*K*_a_ ∼ 6),^53^ strongly adsorbs onto negatively charged surfaces such as glass.^54^ In particular, the herein employed glass supports were cleaned by a UV–O_3_ treatment before printing. After the treatment, the glass surface exhibited highly hydrophilicity, because of the presence of the silanol groups at the interface that are generally mostly deprotonated in water (glass isoelectric point, p*I* ∼ 2).^55^ Then, in order to favor the chitosan adsorption, the pH of the chitosan ink formulation was set at 4, promoting electrostatic polymer–substrate interactions and, simultaneously, ensuring optimal conditions for polymer solubilization. In our experimental conditions, the chitosan ink was formulated in an acidic solution in order to improve its solubility in water by means of the protonation of the amino groups (p*K*_a_ ∼ 6).^56^ The protonation also induces mutual repulsion between the polymeric chains, which results in a good solubilization and in a lowered tendency to aggregate. In addition, the glycerol in the ink formulation contributes to reduce the aggregation phenomena, by interfering with chitosan inter- and intramolecular interactions, thanks to the formation of hydrogen bonds.^40^ Therefore, the formation of supramolecular structures is strongly inhibited. The realization of a homogeneous and continuous chitosan film by inkjet printing required a good inkjettability, which was achieved by means of a conventional jetting operation^41^ (Figure S2a, Supporting Information) and setting the ejection voltage at 40 V. Specifically, the double-pulse waveform, applied to print the chitosan ink, is among the most conventional electrical signal for the formation of droplets by piezoelectric inkjet printing. Whereas the first pulse allows one to produce the pressure wave that results in the liquid thread formation at the nozzle, the second pulse permits one to dampen this oscillating pressure wave, resulting in the droplet pinch-off. The single pulse waveform is typically not enough for the ejection of a well-defined satellite free drop, since it could lead to the production of several satellites as a result of the lack of pressure wave damping.^57^ Furthermore, the printability of chitosan ink in our experimental setup was also evaluated by the estimation of the Ohnesorge numbers (), where μ is the dynamic viscosity of the fluid (mPa·s), ρ is the liquid density (kg/m^3^), σ is the surface tension (mN/m), and *L* is the droplet diameter (μm). From the value of *Oh*, it is possible to calculate the dimensionless printability parameter *Z* (i.e., the inverse of *Oh*).^58^ The fluids whose *Oh* is less than 1 and greater than 0.1 are defined as jettable (i.e., 1 < *Z* < 10). By taking into account the values of viscosity and surface tension reported for the chitosan ink,^59,60^ it was possible to evaluate the printability parameter *Z*, which is ∼4, resulting in a printable ink.^58^ In addition, the experimental conditions used for the droplets inkjetting are both within the printable fluid regime of the Derby plot (Figure S3), further corroborating the good jettability of the two inks. In Figure 2, the stroboscopic images of chitosan ink droplets pinching-off from the nozzle at different times during printing are shown. As it can be observed, the fluid pinched off without forming a detached drop for long times, maintaining its continuity due to the relative high viscosity and surface tension. In the late stages, the drop extended on the vertical axis for several hundred microns and, after the detachment event (e.g., 26 μs), its lengths may arise to the millimeter length scale. The observed elongated tail of the chitosan ink droplets can be attributed to the well-known formation of a long liquid thread (about 800 μm in our case) after droplet pinch-off at the nozzle. This phenomenon is due to the high jetting voltages used for the droplet ejection and has already been observed with different inks containing proteins and biomolecules jetted in the same conditions.^61^ This printing condition was ideal to prevent breakup processes and satellite formations as the drop front was modified only at late stages of the printing (red circles in Figure 2). Indeed as the high viscosity (about 10 mPa s)^60^ significantly dissipates the droplet kinetic energy,^62^ droplet formation was not reproducible at lower jetting voltages, the formed droplets typically pulse at the nozzles by a poor jetting quality. On the other side, the high chitosan surface tension and viscosity allow maintaining the integrity of the jetted droplet at high voltage in agreement with previous studies,^62,63^ by avoiding satellites formation from the elongated tail, as demonstrated by the stroboscopic images of Figure 2. This is corroborated by the fact that aqueous inks characterized by lower viscosity would easily form satellites under high-voltage jetting conditions.^64^ Importantly, the presence of glycerol as an additive in the ink formulation reduces the formation of satellites by stabilizing the liquid thread against capillary instabilities, in accord with previous reports.^65^ Glycerol addition is also meant to avoid aggregation phenomena and to provide longer ink shelf life (>6 months). In fact, glycerol can interfere with chitosan inter- and intramolecular interactions by means of hydrogen bonds formation and stabile chain solvation that strongly reduce the aggregates occurrence.^65^ In addition, the high voltage determined a fast mean speed of the droplet that reached the value of 27.1 ± 0.2 m/s.

As expected, the deposited droplets showed a high level of spreading on the highly hydrophilic glass. Depending on the fixed drop spacing during printing, different patterns can be obtained. As the drop spacing is smaller than the diameter of a sitting droplet (∼100 μm), a complete drops coalescence was achieved, resulting in a liquid ink film and then thermal cured at 100 °C to obtain a thin homogeneous chitosan pad (Figure S2b, Supporting Information). The chitosan layer thickness was estimated to be of the order of 70–80 nm, considering the printed chitosan quantity, its density (0.2–0.3 g/cm^3^),^66^ and the pad area (64 mm^2^). This thickness value is reasonable in comparison with previous chitosan film thickness reports.^67^

Selecting drop spacing larger than 100 μm, the single chitosan spread droplets did not coalesce and were instead patterned in an array fashion (Figure S2c, Supporting Information). Although the polymer coating was employed as first layer for the biochip fabrication, the arrays were also printed to better evaluate the printing quality as well as for fluorescence-based characterization purposes. In fact, the material distribution was more easily investigated exploiting the geometrical features of the array respect to a flat coating. Specifically, chitosan distribution on the printed area was qualitatively evaluated by CLSM. Chitosan patterns were found to absorb fluorescent molecules such as Alexa 647 and interestingly protein molecules such as FITC-BSA (Figure S4, Supporting Information). In both cases, a homogeneous distribution of the biopolymer was observed in correspondence of the printed spots. The fluorescence signal remained stable after several washing cycles. Additionally, the incubation of dry chitosan spots in aqueous solution with dyes induced polymer swelling^68^ that may favor cell adhesion and improve softness at the cell/pattern interface.^69^

### 3.2 Chitosan-*g*-EPEG Characterization

**Figure 3:**
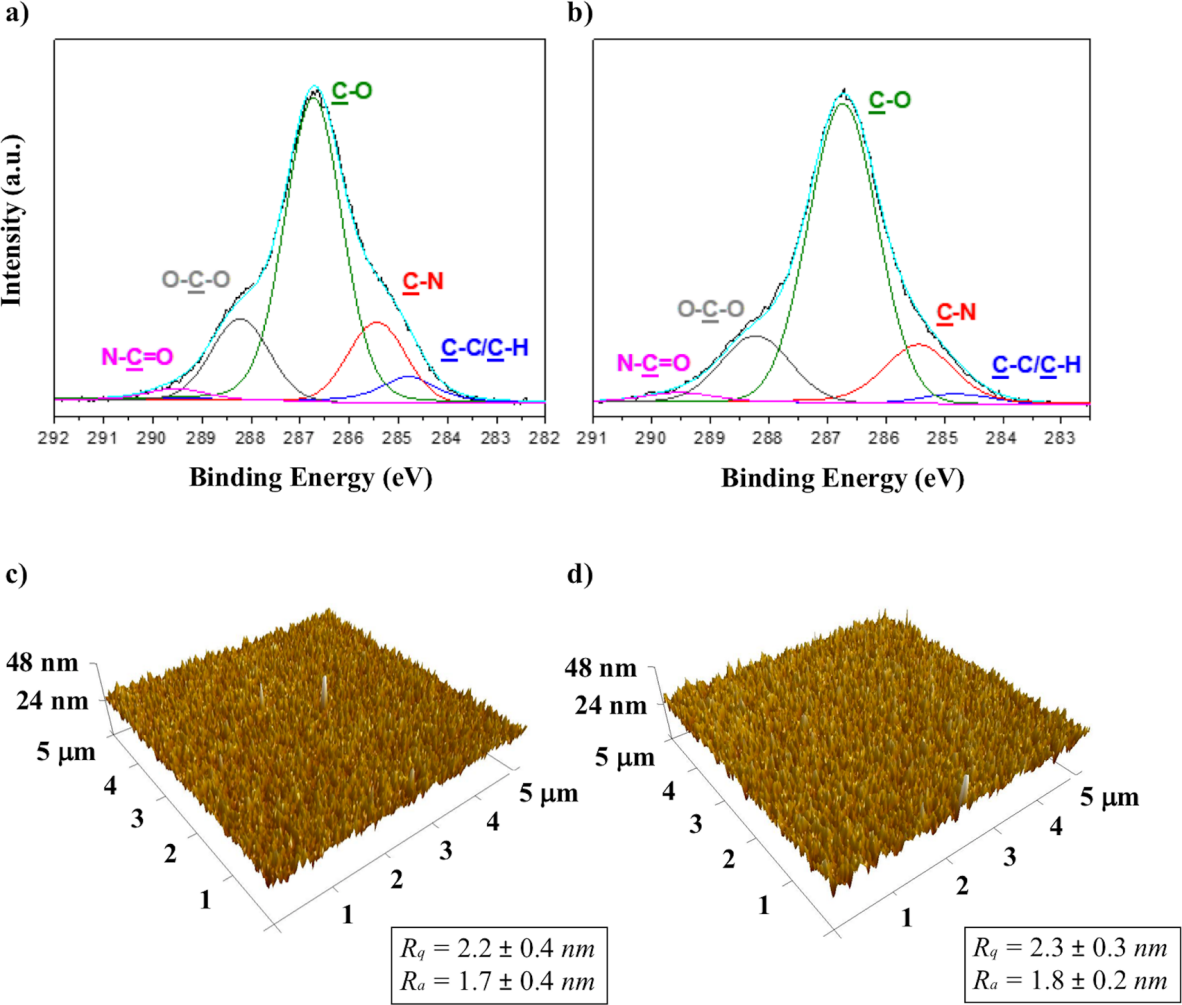
XPS analysis of C 1s peaks for chitosan film before (a) and after (b) EPEG grafting. Tapping mode AFM images of 5 μm × 5 μm area of chitosan film before (c) and after (d) EPEG grafting.

Primary amino groups of the dry printed chitosan film were reacted with the epoxy end groups of EPEG in order to obtain a cell-repellent interface. The grafting reaction consisted in the nucleophilic opening of the epoxide rings by the free chitosan amino groups leading to a stable chemisorption of EPEG.^70^ The reaction was successful carried out in ambient conditions and at room temperature, resulting in the successful formation of the chitosan-*g*-EPEG layer. Surface analysis of the EPEG layer was performed by means of XPS and AFM methods to provide both chemical and morphology properties, respectively. Specifically, survey spectra were recorded for surface elemental analyses. The chitosan film spectrum showed the expected peaks for carbon, nitrogen, and oxygen, with a slight difference between quantitative experimental and theoretical data, likely due to residues of glycerol from the ink and ubiquitous adventitious carbon (Table 1). Additionally, the elemental analyses before and after grafting reaction revealed a similar surface chemistry at the modified and unmodified films surface, dominated by the chitosan chemical composition (Table 1). Since no characteristic element is present in the EPEG structure to distinguish it from chitosan, a further investigation focusing on high resolution C 1s signal, based on the peak components fitting, was necessary. In Figure 3 are shown the high-resolution C 1s peaks fitted with five components corresponding to hydrocarbon bonds C–C/C–H (fixed at a BE of 284.8 eV), carbon-heteroatom single bonds C–N, C–O and O–C–O at 285.4 eV, 286.7eV, and 288.2 eV, respectively, and carbon in amide group N–C=O at 289.5 eV (Figure 3a,b). The EPEG–chitosan surface contains C–O bonds and no aliphatic carbon atoms; therefore, a relative increase in C–O and decrease in C–C/C–H confirmed the reaction (Table 1). The EPEG localization as the top-layer on the chitosan coating will be confirmed by the strongly cell-repulsive properties exhibited by the chitosan-*g*-EPEG film at the biointerface.

**Table 1:** Theoretical and Experimental XPS Average Atomic Composition (%) and Carbon Contents of Different Types of Carbon Chemical Groups (%) from High-Resolution C 1s Peaks^a^

	C	N	O	N–C=O	O–C–O	C–O	C–N	C–C/C–H
chitosan (calculated)	55.0	8.7	36.2					
chitosan	47.6	6.2	39.9	2.0	15.2	60.9	15.2	6.6
chitosan-*g*-EPEG	48.3	5.3	39.9	2.0	14.7	66.5	14.7	2.2

a The theoretical elemental composition of chitosan was calculated by the polymer chemical structure.

The effects of the EPEG grafting on the chitosan film topology and roughness were also investigated by AFM in tapping mode. In Figure 3c,d, representative 5 μm × 5 μm morphology images of the chitosan film before and after the EPEG grafting are reported. The images confirmed the well-film forming properties of chitosan^71^ and the successful process of deposition by inkjet printing. In fact, AFM revealed a continuous and homogeneous coating, suitable for the subsequent fabrication steps. Importantly, the film morphology was not affected by the chemical reaction with EPEG, as shown by the comparable values of both *R*_a_ and *R*_q_ values before and after the functionalization.

### 3.3 Collagen Microarrays Patterning

The collagen printing on the previously described chitosan-*g*-EPEG surface permitted one to define the cell attachment area with resolution at the microscale through an unprecedented printing protocol. In particular, type I collagen ink was dispensed in an array fashion by inkjet printing in order to obtain adhesion spots, whose lateral dimension (diameter ∼32 μm, Figure S5a, Supporting Information) was comparable to the size of a single human adherent cell. The collagen concentration was fixed at 0.08% w/v to improve the material–cell interaction and the yield of cell harvesting. Note that diluted collagen inks reduced the cell attachment and delayed the adhesion kinetics in our experimental conditions, resulting in nonoptimal adhesion (data not shown). The inkjet printing of collagen ink was characterized by two main issues. The first was the relative high concentration of collagen that led to a high viscous fluid prone to aggregation at the nozzles. The second was the necessity to generate droplets small enough (volume ∼10^2^ fL) to produce collagen spots on the highly hydrophilic EPEG interface to fit the lateral size of a single cell. In fact, the hydrophilic EPEG surface induces a high spread of the aqueous inkjetted droplet after impact, increasing the spot diameter and then the number of cells that could attach on a single spot.

As with the chitosan ink, the collagen tendency to aggregation and the ink printability were significantly improved by introducing glycerol as a cosolvent. Furthermore, glycerol plays an additional role in the collagen ink formulation, as a stabilizing agent for the collagen triple-helix structure, through the formation of multiple hydrogen bonds, replacing the tightly bound hydration water molecules.^72^ This process limits the aggregation of collagen in the cartridge during printing as well as during the storage, resulting in a longer ink shelf life (>3 months at 4 °C). Analogously to chitosan ink, the collagen ink printability was evaluated by the *Oh* number calculation based on the rheological properties of the ink.^73,74^ The value *Z* for collagen is ∼8, resulting in a well-printable ink (Figure S3).^58^

**Figure 4:**
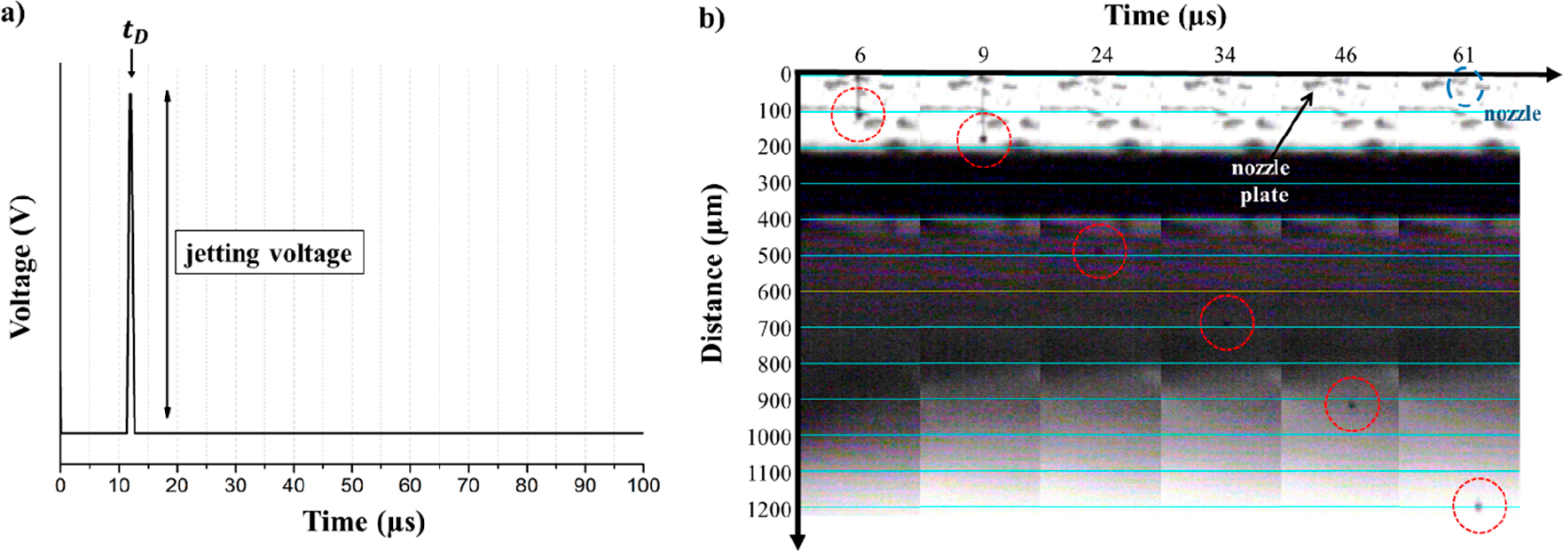
Printing condition for collagen ink ejection: (a) single short-pulse waveform optimized for aqueous inkjet printing at the femtoliter-scale with a pulse length of 1.3 μs and (b) stroboscopic images of collagen ink droplets ejected by a nozzle of a diameter of 10.5 μm at 30 V jetting voltage, recorded from 6 to 61 μs.

The collagen spot lateral size was tuned by experimentally reproducing the Eggers’s theoretical model,^75^ which demonstrates that a fine-tuning of the inkjetted droplet volume can be achieved by varying the droplet emission time.^76^ To this aim, the waveform in Figure 4a was optimized and adapted for inkjet printing of aqueous inks at the femtoliter-scale. It consists in a short-pulse waveform developed to print aqueous droplets by a conventional 1 pL-ejecting cartridge with nozzle diameters of 10.5 μm, which enabled the formation of a droplet smaller than the nozzle size. As reported in our previous investigations, it is possible to produce subnozzle sized droplets by minimizing the *t*_D_.^76^ The mechanism for this process is based on the theoretical model described by Eggers, which in turn relies on a singularity of the Navier–Stokes equations describing the droplet size at the initial formation stages.^75^ This short pulse waveform permitted to obtain droplets which did not show any tail, as a consequence of the reduced volume jetted at the nozzle which, in turn, simply led to almost spherical droplets, in accordance with our previous investigations on aqueous inks jetting at femtoliter-scale volumes.^76^ Differently to the chitosan ink, the collagen ink was fairly printable by this waveform at jetting voltages comprised between 30–40 V. It was not possible to print at voltages lower than 30 V. This can be again likely explained by considering the ink viscosity (3 mPa s) and the high surface tension (70 mN/m) which dissipate the droplet kinetic energy during its formation at the nozzles.^62^Figure 4b shows the stroboscopic images of collagen ink droplets ejected by applying such a waveform at 30 V jetting voltage after droplet formation at the nozzle. It was verified that the developed waveform allowed one to print tiny and spherical droplets of collagen ink with no satellite formations and optimal directionality. The sizes of the collagen droplets were qualitatively estimated by considering the stroboscopic images of the droplets formed at the nozzle.

**Figure 5:**
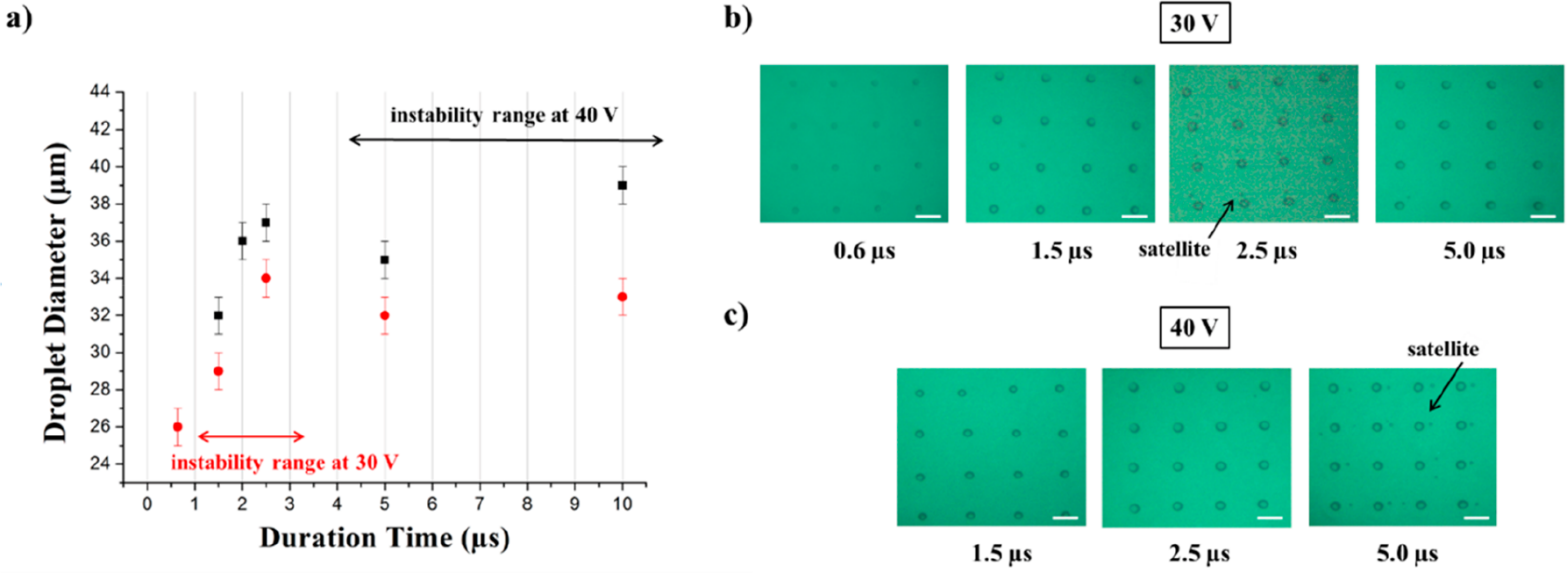
Effect of the *t*_D_ on the dimension of sessile droplets of 0.08% w/v collagen ink: (a) representative sessile droplet size for each *t*_D_, ejected at 30 V (red) and 40 V (black). The error bars represent the experimental error associated with the diameter measurement via optical microscopy; (b) white-field images of droplets microarrays printed at 40 V and (c) at 30 V. The graph in panel a also shows printing instability regions (double arrows), where breakup phenomena induce droplet fragmentation and satellites formation, as shown in the corresponding pictures of wet droplets arrays (satellites are indicated by arrows in panels b and c). Scale bars 100 μm.

The jetting voltage and the time of application of the highest value of the electric pulse (duration time, *t*_D_) were investigated in order to optimize the sessile droplets dimension to fit the single cell size. In particular, the short-pulse waveform was applied varying the *t*_D_ in the range comprised between 0.6 and 23.0 μs at different voltage values (30 and 40 V) resulting in collagen arrays with different spot diameters, and the results are reported in Figure 5. As evident in Figure 5a, where the droplet diameters are reported as a function of experimental conditions, larger lateral dimensions were obtained at higher applied voltage.

Moreover, in accordance with previous data,^76^ the resulting sessile droplet diameter was found to increase at longer *t*_D_, reaching a plateau at *t*_D_ > 4.0 μs. This effect is likely due to the viscosity of the ink that reduces the further droplet size increase. Notably, the emergence of capillary instability in the ejected liquid thread triggered droplet breakups regimes and poor directionality, which were markedly depending on the applied jetting voltage. Representative images of the sessile droplets printed on the hydrophilic EPEG surfaces at 30 and 40 V are shown in Figure 5b,c, respectively. The diameter distribution of the sessile droplets is reported in Figure S6 (Supporting Information). Different instability ranges can be explained in terms of the fluid dynamics of the ink ejection. For the droplets ejected at 30 V, at *t*_D_ shorter than 1.0 μs, the droplets were small enough to approximate the Eggers’s conditions, reaching subpicoliter scale diameter. Interestingly, at this voltage the capillary instability region occurred at shorter *t*_D_ (1.0–4.0 μs), because of the low droplet velocity during the ejection, which determines low directionality and higher probabilities of fragmentation. At *t*_D_ > 4.0 μs, the droplet speed is high enough (>10 m/s) to improve its directionality and avoid satellites on the surface.

Differently, the droplets printed at 40 V were characterized by instability and fragmentation phenomena at longer *t*_D_ (>4.0 μs), generally due to the larger dimensions of the drops, that in the first phase of the pinching-off showed an elongated shape, which is known to facilitate the breakup phenomena, leading to satellite formation.^77^ In the next section, it will be shown that the best experimental conditions to fit the single-cell array formation with the higher yield correspond to the short-pulse waveform with a *t*_D_ of 5.0 μs applied at 30 V. This voltage, which is relatively high with respect to commonly used printing conditions, led to a mean velocity of (20.0 ± 0.5) m/s for collagen ink droplets.

As above-mentioned, the optimized collagen ink was printed onto the EPEG functionalized surface, on which unreacted epoxide groups were exposed at the interface. Specifically, the epoxy groups on chitosan-*g*-EPEG coating were exploited for collagen immobilization by chemisorption of the protein triple-helix, through the reaction between the epoxide rings and the nucleophilic groups at the amino acid residues side chains. As in the case of chitosan patterns, the collagen microarrays were aspecifically stained and imaged by CLSM (Figure S5b, Supporting Information). Sypro Orange was employed as a fluorescent staining agent to demonstrate the possibility to fluoro-label the collagen microarray by means of a hydrophobic dye which present a larger affinity for collagen with respect to chitosan. The staining permitted to evaluate the material distribution in correspondence to the dry collagen spots that appeared circular and properly arranged in the array. The spot morphology was characterized by a central core of biopolymer clump with a surrounding annulus of lower fluorescence. No evidence of coffee-ring effects was observed after drying of the femtoliter-droplets, due to the predominance of the fast liquid evaporation with respect to the radial capillary flow. The collagen distribution on the spot area and its accumulation at the central zone suggested a constant contact-angle evaporation mode,^78^ where the liquid/solid contact surface recedes, carrying protein aggregates at the spot central area. By considering the printed quantity, the typical collagen density (around 1.3 g/cm^3^),^79^ and the spot area values, the collagen spot thickness has been evaluated to be of the order of 50–60 nm, in good accord with reported thickness collagen films thickness values (i.e., around 40 nm).^80^

### 3.4 Single-Cell Array

**Figure 6:**
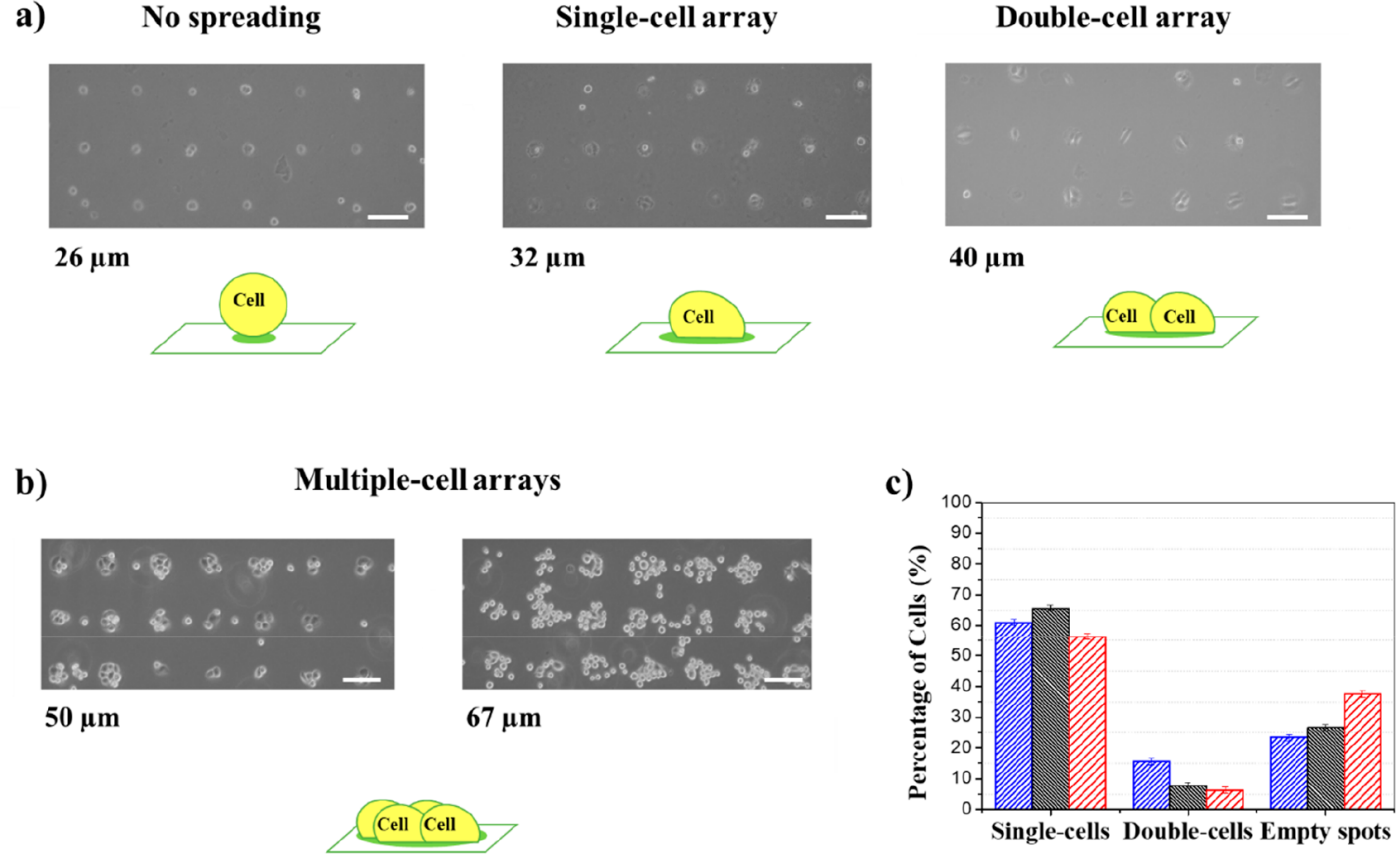
H1975 cell array on collagen patterns of different spot sizes. The collagen arrays in panel a were printed by a 1 pL ejecting-cartridge at *t*_D_ values of 0.6, 5.0, and 10.0 μs (left to right), while the arrays in panel b were printed by a 10 pL ejecting-cartridge at *t*_D_ values of 10.0 and 23 μs (left to right). The number of cells per spot clearly increases with the spot diameter, starting from a nonspreading condition (22 μm) to obtain cell consortia patterns (50–67 μm). Scale bars 50 μm. The single-cell yield (panel c) was calculated on 8 × 8 spots collagen microarrays (spot diameter 32 μm). The mean percentages were calculated using three replicate samples. Error bars indicate the standard deviation. The biological evolution up to 48 h after the adhesion, distinguished in single-cells, double-cells, and empty spots, counted at 1 h (blue), 24 h (black), and 48 h (red) after the adhesion.

Inkjet-printed collagen microarrays were employed as functional supports for culture of human nonsmall-cell lung cancer (NSCLC) cell line H1975. In Figure 6, representative optical images of cell cultured on different collagen microarrays obtained by tuning jetting voltage and *t*_D_ are presented. As shown, the spot diameter strongly determined the area suitable for the cell attachment, and as a consequence of the collagen spot size, the cell spatial arrangement changed along with the number of cells found on a single spot. The cell number and harvesting efficiency was investigated as a function of the collagen-rich spot diameter, in the range from ∼26 μm to ∼67 μm. A 1 pL nozzle was used to obtain diameters up to 40 μm (Figure 6a), while larger spots were obtained by printing the collagen ink using 10 pL nozzles (Figure 6b). The most regular arrays were tested as attachment platforms in order to find the best printing conditions for a single-cell array formation. For all investigated patterns, collagen at the interface triggered the cell array formation with a high yield in terms of cell adhesion (>76% of occupied spots).

Cells accommodated on the patterns following the spots arrangement and geometry and extending to the larger accessible surface, a process in line with previous results, indicate that cells adapt their morphology with dependence on the adhesion pattern geometry and shape.^81^

The smallest spots (∼26 μm) led to a high yield in terms of single-cell, which was ∼100%. However, due to the reduced accessible area for the adhesion, cells could not spread and most of them detached off from the array in an overnight incubation. For collagen spots with diameters larger than ∼30 μm, optimal spreading of cells is always observed as well as stable adhesion for at least for 1 week. As can be seen, arrays with ∼32 μm diameter spots were permitted to realize a microarray of single-cells showing optimal spreading. A further increase to ∼40 μm corresponded to mainly double-cell arrays. In the case of the larger diameters of ∼50 μm and ∼67 μm, groups of cells colonizing a single spot were observed, as expected for larger spot sizes. This effect could also be ascribed to the increase of the collagen spot thickness that is proportional to the droplet diameter in a high spreading condition.^82^ Therefore, the screening highlighted that printing collagen spots with a diameter of ∼32 μm allowed inducing the formation arrays of individual cells with optimal adhesion and spreading. Concerning the adhesion kinetics, after seeding cells on the collagen pattern, the single-cell array was readily formed in about 1 h. As reported in Figure 6c, the yield in terms of the single cell reached 62% after 1 h, and afterward the system further evolved reaching the 66% of single-cells after 24 h. The variation of the single-cells percentageon the array is probably related to the spreading of one cell on a double-occupied spot: one of the cells in a couple likely spreads reducing the available adhesion area for the second anchored cell, which detaches from the support. After 48 h, a slight general loss of cells from the platform reduced the single-cell percentage at 56%. The corresponding analysis in terms of number of cells is also reported to show the amount of cells involved in the experiments (Figure S7, Supporting Information). Remarkably, slightly increasing the collagen spot diameter to 40 μm led to double-cells array, supporting the idea that the optimal accessible collagen surface tunes the cell positioning in a controlled fashion.

**Figure 7:**
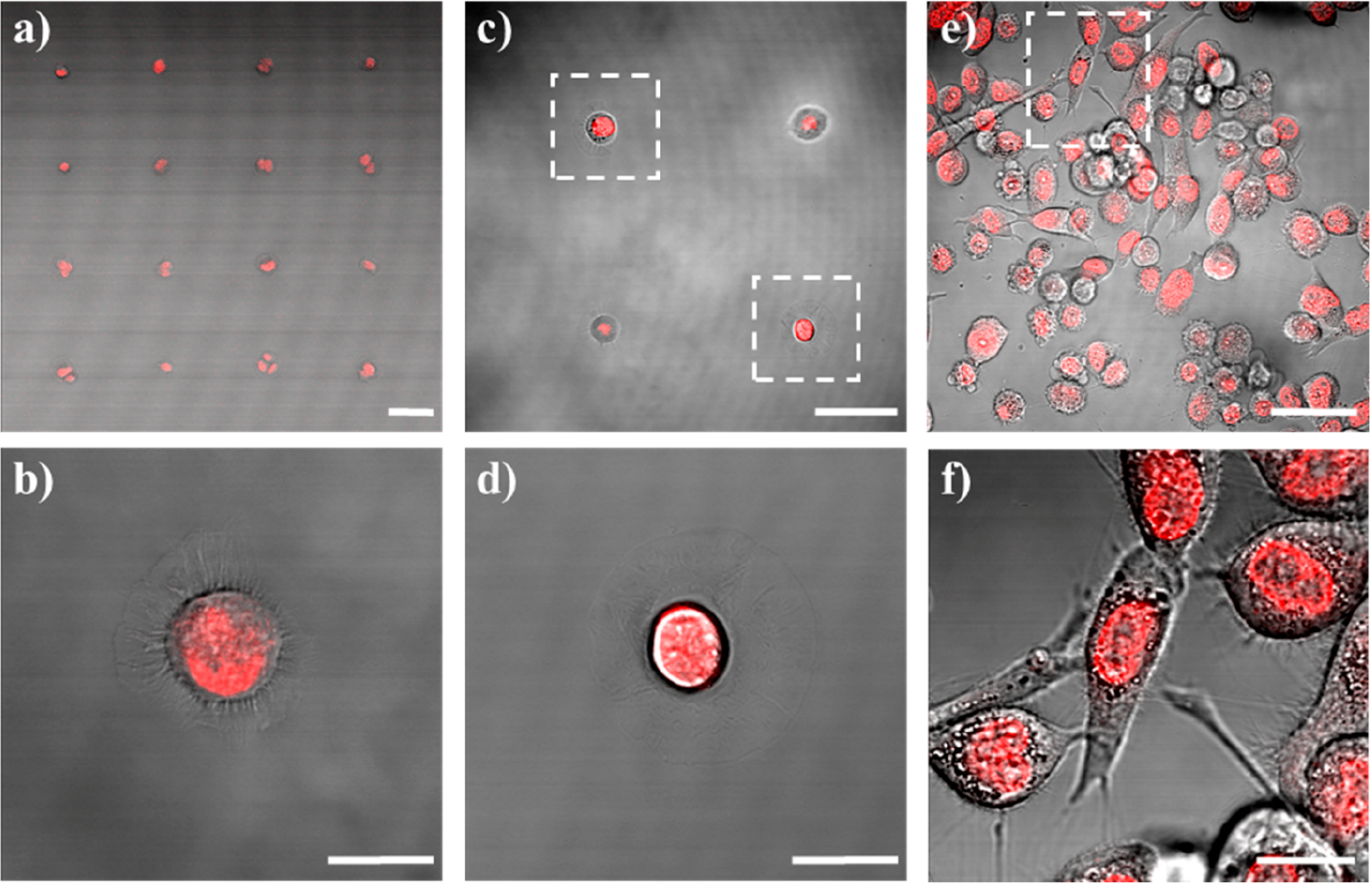
Representative fluorescence images of Dox (red) intracellular localization after 1 h in H1975 cells overlaid on the transmission channel (gray) to show complete cell morphology: (a–d) single-cell arrays and (e,f) conventional cell culture. Framed cells in panels c and e are shown in panels b,d, and f, respectively. Scale bars 20 μm in panels a–d, 50 μm in panels e and f.

Finally, it was interesting to provide a proof-of-concept pharmacological treatment on the developed single-cell platform to assess the feasibility of calibrated single cell biology experiments. Specifically, in order to better understand the response to chemical stimuli on the captured human cancer cells, the uptake of Dox, a widely used and effective drug for the treatment of a plethora of solid human cancers,^83^ was investigated. CLSM was used to detect Dox fluorescence signal in order to verify the drug internalization and its nuclear localization. In Figure 7, representative fluorescence confocal images overlapped with transmission pictures of H1975 single-cell arrays treated with Dox 100 μM for 1 h are reported (panels a–d) along with corresponding conventional cell culture treated in the same way as the reference (panels f and g). The fluorescence signal (red) was attributed to Dox and was found in almost all cells reported. As shown in the higher resolution images, Dox localized in the nuclei both for cells in arrays and in standard cultures.

## 4 Conclusions

The herein presented work shows an innovative, aqueous-processed, green-chemistry printing approach for the fabrication of new polymeric platforms able to control cell localization on solid supports, with the aim to obtain cell arrays with a controllable number of cells on each spot, ranging from cells consortia to single-cell arrays with a high yield. The whole fabrication protocol involves aqueous inks containing biocompatible polymers, such as chitosan, EPEG, and type I collagen. No organic solvents or cytotoxic chemicals were introduced, resulting in an all-aqueous green fabrication strategy, in line with the principles of green chemistry.^84^ Then, the herein presented biochip represents an example of all-aqueous single-cell array obtained by piezoelectric inkjet printing, opening up new perspectives for innovative eco-friendly strategies in this field. Additionally, the developed approach is characterized by a remarkable versatility in terms of printed spot size that permits one to span a large range of the biomaterial pattern dimensions, simply by tuning the printing waveform duration time at the microsecond time-scale. This constitutes a relevant advantageous aspect with respect to other deposition strategies as the scanning probe lithography methods, whose patterning time scales are on the order of seconds to span in the micron-scale feature ranges. It is noteworthy that the high-throughput capability of the piezoelectric inkjet printing is among the most relevant features of this technology. In fact, in order to increase the printing speed, it would be possible to simultaneously use multiple nozzles enabling the inkjet printing technology for large scale industrial applications.^85^

In turn, the fine control on the spots size allowed tuning the extent of the collagen attachment area useful for cell capture, then arrays of single, double, and multiple cells can be achieved. The capability to modify the number of cells per spot highlights the possibility to employ the herein presented platform for cellular investigations at different levels of details, from the extraction of peculiar biological properties of the individual cells in a single-cell array to cellular communication studies in double- and multiple-cell arrays with a tunable number of cells per spot, aspects of great relevance to unravel the complex mechanisms involved in tumor and inflammatory processes. In addition, the optimization of the collagen spot dimensions leads also to obtain spots that best fit the conditions for patterning cells able to spread on the support. In fact, it was possible to observe functional single human lung cancer cells on arrays of ∼32 μm diameter collagen spots. Notably, the efficient adhesion of the cells on the spots is fundamental to carry out reliable biological studies on living cell platforms, and it has not been taken into account in many relevant single-cell arrays fabricated through different approaches.^19,86,87^ The platform was developed on glass, allowing for microscopic analyses by fluorescence confocal microscopy. The efficient internalization of the anticancer drug doxorubicin is a proof-of-concept to investigate the platform influence on cell behavior, demonstrating the absence of any device-related effects concerning drug internalization process. Focusing on the single-cell array, it easily offers the possibility to analyze individual cell as independent samples, toward more consistent and robust statistical analyses and dramatic reduction in terms of the number of experiments to carry out. These findings represent a first step toward the integration of biopolymeric interfaces for the fabrication of single-cell biochip devices.

Finally, the main goal of the herein shown platforms is their application as lab-on-a-chip devices for detailed investigations of cellular processes at the single-cell level with a robust statistical approach, since each spot on the support can be envisaged as an individual sample. Moreover, by following the 3D printing developments aiming to engineering artificial tissues, the presented approach might allow the reconstruction of 2D tissue sections mimicking the tissue architecture by a very high spatial resolution (single/multiple cells level). In principle, the here presented platform can find immediate applications in the field of biopolymers-based scaffolds, providing a highly biocompatible environment for cells attachment and growth.^48,88^ Remarkably, our novel ecofriendly manufacturing approach might solve the issues of toxic solvents utilization in the fabrication of polymer-based scaffolds for tissue engineering, which is often encountered for instance within electrospinned polymer-based scaffolds for tissue regeneration.^89^

## Supporting Information Available

Biochip architecture, printing conditions for chitosan ink ejection, Derby plot for the chitosan and collagen inks, fluorescence images of chitosan patterns, collagen microarrays staining, diameter distributions of collagen ink sessile droplets, and count of cells adherent on collagen array (PDF)

## References

[ref1] Schwartzman O.; Tanay A. Single-Cell Epigenomics: Techniques and Emerging Applications. Nat. Rev. Genet. 2015, 16, 716–726. 10.1038/nrg3980. 26460349

[ref2] Stegle O.; Teichmann S. A.; Marioni J. C. Computational and Analytical Challenges in Single-Cell Transcriptomics. Nat. Rev. Genet. 2015, 16, 133–145. 10.1038/nrg3833. 25628217

[ref3] Niu F.; Wang D. C.; Lu J.; Wu W.; Wang X. Potentials of Single-Cell Biology in Identification and Validation of Disease Biomarkers. J. Cell. Mol. Med. 2016, 20 (9), 1789–1795. 10.1111/jcmm.12868. 27113384 PMC4988278

[ref4] Litzenburger U. M.; Buenrostro J. D.; Wu B.; Shen Y.; Sheffield N. C.; Kathiria A.; Greenleaf W. J.; Chang H. Y. Single-Cell Epigenomic Variability Reveals Functional Cancer Heterogeneity. Genome Biol. 2017, 18 (1), 15 10.1186/s13059-016-1133-7. 28118844 PMC5259890

[ref5] Heath J. R.; Ribas A.; Mischel P. S. Single-Cell Analysis Tools for Drug Discovery and Development. Nat. Rev. Drug Discovery 2016, 15 (3), 204–216. 10.1038/nrd.2015.16. 26669673 PMC4883669

[ref6] Dittrich P. S.; Manz A. Lab-on-a-Chip: Microfluidics in Drug Discovery. Nat. Rev. Drug Discovery 2006, 5 (3), 210–218. 10.1038/nrd1985. 16518374

[ref7] Raj A.; van Oudenaarden A. Nature, Nurture, or Chance: Stochastic Gene Expression and Its Consequences. Cell 2008, 135 (2), 216–226. 10.1016/j.cell.2008.09.050. 18957198 PMC3118044

[ref8] Gandor S.; Reisewitz S.; Venkatachalapathy M.; Arrabito G.; Reibner M.; Schröder H.; Ruf K.; Niemeyer C. M.; Bastiaens P. I. H.; Dehmelt L. A Protein-Interaction Array Inside a Living Cell. Angew. Chem., Int. Ed. 2013, 52 (18), 4790–4794. 10.1002/anie.201209127. PMC3652028 23460583

[ref9] Yan R.; Park J. H.; Choi Y.; Heo C. J.; Yang S. M.; Lee L. P.; Yang P. Nanowire-Based Single-Cell Endoscopy. Nat. Nanotechnol. 2012, 7 (3), 191–196. 10.1038/nnano.2011.226. 22179570

[ref10] Errico V.; Arrabito G.; Fornetti E.; Fuoco C.; Testa S.; Saggio G.; Rufini S.; Cannata S.; Desideri A.; Falconi C.; Gargioli C. High-Density ZnO Nanowires as a Reversible Myogenic–Differentiation Switch. ACS Appl. Mater. Interfaces 2018, 10 (16), 14097–14107. 10.1021/acsami.7b19758. 29619824

[ref11] Errico V.; Arrabito G.; Plant S. R.; Medaglia P. G.; Palmer R. E.; Falconi C. Chromium Inhibition and Size-Selected Au Nanocluster Catalysis for the Solution Growth of Low-Density ZnO Nanowires. Sci. Rep. 2015, 5, 12336 10.1038/srep12336. 26202588 PMC4511950

[ref12] Yamamura S.; Kishi H.; Tokimitsu Y.; Kondo S.; Honda R.; Rao S. R.; Omori M.; Tamiya E.; Muraguchi A. Single-Cell Microarray for Analyzing Cellular Response. Anal. Chem. 2005, 77, 8050–8056. 10.1021/ac0515632. 16351155

[ref13] Wlodkowic D.; Faley S.; Zagnoni M.; Wikswo J. P.; Cooper J. M. Microfluidic Single-Cell Array Cytometry for the Analysis of Tumor Apoptosis. Anal. Chem. 2009, 81 (13), 5517–5523. 10.1021/ac9008463. 19514700 PMC3816605

[ref14] Zheng X.; Pan X.; Pang Q.; Shuai C.; Ma L.; Gao C. Selective Capture of Mesenchymal Stem Cells over Fibroblasts and Immune Cells on E7-Modified Collagen Substrates under Flow Circumstances. J. Mater. Chem. B 2018, 6 (1), 165–173. 10.1039/C7TB02812A. 32254204

[ref15] Yoon H. J.; Kim T. H.; Zhang Z.; Azizi E.; Pham T. M.; Paoletti C.; Lin J.; Ramnath N.; Wicha M. S.; Hayes D. F.; Simeone D. M.; Nagrath S. Sensitive Capture of Circulating Tumour Cells by Functionalized Graphene Oxide Nanosheets. Nat. Nanotechnol. 2013, 8 (10), 735–741. 10.1038/nnano.2013.194. 24077027 PMC4017624

[ref16] Wang Z.; Zhang P.; Kirkland B.; Liu Y.; Guan J. Microcontact Printing of Polyelectrolytes on PEG Using an Unmodified PDMS Stamp for Micropatterning Nanoparticles, DNA, Proteins and Cells. Soft Matter 2012, 8 (29), 7630–7637. 10.1039/c2sm25835h.

[ref17] Collins J. M.; Nettikadan S. Subcellular Scaled Multiplexed Protein Patterns for Single Cell Cocultures. Anal. Biochem. 2011, 419 (2), 339–341. 10.1016/j.ab.2011.08.021. 21907699

[ref18] Qiao Y.; Ma L. Quantification of Metal Ion Induced DNA Damage with Single Cell Array Based Assay. Analyst 2013, 138 (19), 5713 10.1039/c3an00967j. 23892322

[ref19] Collins J. M.; Lam R. T. S.; Yang Z.; Semsarieh B.; Smetana A. B.; Nettikadan S. Targeted Delivery to Single Cells in Precisely Controlled Microenvironments. Lab Chip 2012, 12 (15), 2643–2648. 10.1039/c2lc40216e. 22622356

[ref20] Vermesh U.; Vermesh O.; Wang J.; Kwong G. A.; Ma C.; Hwang K.; Heath J. R. High-Density, Multiplexed Patterning of Cells at Single-Cell Resolution for Tissue Engineering and Other Applications. Angew. Chem., Int. Ed. 2011, 50 (32), 7378–7380. 10.1002/anie.201102249. PMC3651859 21717543

[ref21] Arrabito G.; Reisewitz S.; Dehmelt L.; Bastiaens P. I.; Pignataro B.; Schroeder H.; Niemeyer C. M. Biochips for Cell Biology by Combined Dip-Pen Nanolithography and DNA-Directed Protein Immobilization. Small 2013, 9 (24), 4243–4249. 10.1002/smll.201300941. 23881817

[ref22] Li H.; Yang Q.; Li G.; Li M.; Wang S.; Song Y. Splitting a Droplet for Femtoliter Liquid Patterns and Single Cell Isolation. ACS Appl. Mater. Interfaces 2015, 7 (17), 9060–9065. 10.1021/am509177s. 25761507

[ref23] Gudapati H.; Dey M.; Ozbolat I. A Comprehensive Review on Droplet-Based Bioprinting: Past, Present and Future. Biomaterials 2016, 102, 20–42. 10.1016/j.biomaterials.2016.06.012. 27318933

[ref24] Arrabito G.; Galati C.; Castellano S.; Pignataro B. Luminometric Sub-Nanoliter Droplet-to-Droplet Array (LUMDA) and Its Application to Drug Screening by Phase I Metabolism Enzymes. Lab Chip 2013, 13 (1), 68–72. 10.1039/C2LC40948H. 23132304

[ref25] Arrabito G.; Pignataro B. Solution Processed Micro- and Nano-Bioarrays for Multiplexed Biosensing. Anal. Chem. 2012, 84 (13), 5450–5462. 10.1021/ac300621z. 22591457

[ref26] Guo J.; Ling S.; Li W.; Chen Y.; Li C.; Omenetto F. G.; Kaplan D. L. Coding Cell Micropatterns Through Peptide Inkjet Printing for Arbitrary Biomineralized Architectures. Adv. Funct. Mater. 2018, 28 (19), 1800228 10.1002/adfm.201800228. 32440260 PMC7241601

[ref27] Tourniaire G.; Collins J.; Campbell S.; Mizomoto H.; Ogawa S.; Thaburet J. F.; Bradley M. Polymer Microarrays for Cellular Adhesion. Chem. Commun. 2006, (20), 2118–2120. 10.1039/b602009g. 16703126

[ref28] Li J.; Rossignol F.; Macdonald J. Lab on a Chip Inkjet Printing for Biosensor Fabrication: Combining Chemistry and Technology for Inkjet Printers. Lab Chip 2015, 15, 2538–2558. 10.1039/C5LC00235D. 25953427

[ref29] Park J. A.; Yoon S.; Kwon J.; Now H.; Kim Y. K.; Kim W.; Yoo J.; Jung S. Freeform Micropatterning of Living Cells into Cell Culture Medium Using Direct Inkjet Printing. Sci. Rep. 2017, 7 (May), 1–11. 10.1038/s41598-017-14726-w. 29097768 PMC5668285

[ref30] Kim B. S.; Lee J.; Gao G.; Cho D. Direct 3D Cell-Printing of Human Skin with Functional Transwell System Direct 3D Cell-Printing of Human Skin with Functional Transwell System. Biofabrication 2017, 9, 025034 10.1088/1758-5090/aa71c8. 28586316

[ref31] Calvert P. Printing Cells. Science 2007, 318 (5848), 208–209. 10.1126/science.1144212. 17932278

[ref32] Roth E. A.; Xu T.; Das M.; Gregory C.; Hickman J. J.; Boland T. Inkjet Printing for High-Throughput Cell Patterning. Biomaterials 2004, 25 (17), 3707–3715. 10.1016/j.biomaterials.2003.10.052. 15020146

[ref33] Sanjana N. E.; Fuller S. B. A Fast Flexible Ink-Jet Printing Method for Patterning Dissociated Neurons in Culture. J. Neurosci. Methods 2004, 136 (2), 151–163. 10.1016/j.jneumeth.2004.01.011. 15183267

[ref34] Sun Y.; Song W.; Sun X.; Zhang S. Inkjet-Printing Patterned Chip on Sticky Superhydrophobic Surface for High-Efficiency Single-Cell Array Trapping and Real-Time Observation of Cellular Apoptosis. ACS Appl. Mater. Interfaces 2018, 10 (37), 31054–31060. 10.1021/acsami.8b10703. 30148358

[ref35] Jang J.-W.; Collins J. M.; Nettikadan S. User-Friendly Universal and Durable Subcellular-Scaled Template for Protein Binding: Application to Single-Cell Patterning. Adv. Funct. Mater. 2013, 23 (47), 5840–5845. 10.1002/adfm.201301088.

[ref36] Arrabito G.; Cavaleri F.; Montalbano V.; Vetri V.; Leone M.; Pignataro B. Monitoring Few Molecular Binding Events in Scalable Confined Aqueous Compartments by Raster Image Correlation Spectroscopy (CADRICS). Lab Chip 2016, 16 (24), 4666–4676. 10.1039/C6LC01072E. 27812580

[ref37] Arrabito G.; Ferrara V.; Ottaviani A.; Cavaleri F.; Cubisino S. A. M.; Cancemi P.; Ho Y.-P.; Knudsen B. R.; Hede M. S.; Pellerito C.; Desideri A.; Feo S.; Pignataro B. Imbibition of FL-Scale DNA-Rich Aqueous Droplets into Porous Nylon Substrates by Molecular Printing. Langmuir 2019, 35 (52), 17156–17165. 10.1021/acs.langmuir.9b02893. 31790261

[ref38] Cole R. H.; Tang S.-Y.; Siltanen C. A.; Shahi P.; Zhang J. Q.; Poust S.; Gartner Z. J.; Abate A. R. Printed Droplet Microfluidics for on Demand Dispensing of Picoliter Droplets and Cells. Proc. Natl. Acad. Sci. U. S. A. 2017, 114 (33), 1–6. 10.1073/pnas.1704020114. PMC5565430 28760972

[ref39] Qiao Y.; Zhou Y.; Xiao T.; Zhang Z.; Ma L.; Su M.; Suo G. Evaluating Single-Cell DNA Damage Induced by Enhanced Radiation on a Gold Nanofilm Patch. ACS Appl. Mater. Interfaces 2017, 9 (42), 36525–36532. 10.1021/acsami.7b08460. 28984132

[ref40] Rivero S.; Damonte L.; García M. A.; Pinotti A. An Insight into the Role of Glycerol in Chitosan Films. Food Biophys. 2016, 11 (2), 117–127. 10.1007/s11483-015-9421-4.

[ref41] Arrabito G.; Cavaleri F.; Montalbano V.; Vetri V.; Leone M.; Pignataro B. Monitoring Few Molecular Binding Events in Scalable Confined Aqueous Compartments by Raster Image Correlation Spectroscopy (CADRICS). Lab Chip 2016, 16 (24), 4666–4676. 10.1039/C6LC01072E. 27812580

[ref42] Constantine C. A.; Mello S. V.; Dupont A.; Cao X.; Santos D.; Oliveira O. N.; Strixino F. T.; Pereira E. C.; Cheng T.-C.; Defrank J. J.; Leblanc R. M. Layer-by-Layer Self-Assembled Chitosan/Poly(Thiophene-3-Acetic Acid) and Organophosphorus Hydrolase Multilayers. J. Am. Chem. Soc. 2003, 125 (7), 1805–1809. 10.1021/ja028691h. 12580606

[ref43] Crivello J. V.; Liu S. Photoinitiated Cationic Polymerization of Epoxy. J. Polym. Sci., Part A: Polym. Chem. 2000, 38 (May), 389–401. 10.1002/(SICI)1099-0518(20000201)38:3<389::AID-POLA1>3.0.CO;2-G.

[ref44] Herzberger J.; Niederer K.; Pohlit H.; Seiwert J.; Worm M.; Wurm F. R.; Frey H. Polymerization of Ethylene Oxide, Propylene Oxide, and Other Alkylene Oxides: Synthesis, Novel Polymer Architectures, and Bioconjugation. Chem. Rev. 2016, 116 (4), 2170–2243. 10.1021/acs.chemrev.5b00441. 26713458

[ref45] Arrabito G.; Schroeder H.; Schröder K.; Filips C.; Marggraf U.; Dopp C.; Venkatachalapathy M.; Dehmelt L.; Bastiaens P. I. H.; Neyer A.; Niemeyer C. M. Configurable Low-Cost Plotter Device for Fabrication of Multi-Color Sub-Cellular Scale Microarrays. Small 2014, 10 (14), 2870–2876. 10.1002/smll.201303390. 24678019

[ref46] Gelse K.; Pöschl E.; Aigner T. Collagens - Structure, Function, and Biosynthesis. Adv. Drug Delivery Rev. 2003, 55 (12), 1531–1546. 10.1016/j.addr.2003.08.002. 14623400

[ref47] De Vries J. W.; Zhang F.; Herrmann A. Drug Delivery Systems Based on Nucleic Acid Nanostructures. J. Controlled Release 2013, 172 (2), 467–483. 10.1016/j.jconrel.2013.05.022. 23742878

[ref48] Preethi Soundarya S.; Haritha Menon A.; Viji Chandran S.; Selvamurugan N. Bone Tissue Engineering: Scaffold Preparation Using Chitosan and Other Biomaterials with Different Design and Fabrication Techniques. Int. J. Biol. Macromol. 2018, 119, 1228–1239. 10.1016/j.ijbiomac.2018.08.056. 30107161

[ref49] Wang H.; Qian J.; Ding F. Emerging Chitosan-Based Films for Food Packaging Applications. J. Agric. Food Chem. 2018, 66 (2), 395–413. 10.1021/acs.jafc.7b04528. 29257871

[ref50] Ligler F. S.; Lingerfelt B. M.; Price R. P.; Schoen P. E. Development of Uniform Chitosan Thin-Film Layers on Silicon Chips. Langmuir 2001, 17 (16), 5082–5084. 10.1021/la010148b.

[ref51] Gu Y.; Zhang W.; Wang H.; Lee W. Y. Chitosan Surface Enhances the Mobility, Cytoplasm Spreading, and Phagocytosis of Macrophages. Colloids Surf., B 2014, 117, 42–50. 10.1016/j.colsurfb.2014.01.051. 24632029

[ref52] Caro N.; Medina E.; Díaz-Dosque M.; López L.; Abugoch L.; Tapia C. Novel Active Packaging Based on Films of Chitosan and Chitosan/Quinoa Protein Printed with Chitosan-Tripolyphosphate-Thymol Nanoparticles via Thermal Ink-Jet Printing. Food Hydrocolloids 2016, 52, 520–532. 10.1016/j.foodhyd.2015.07.028.

[ref53] Rabea E. I.; Badawy M. E. T.; Stevens C. V.; Smagghe G.; Steurbaut W. Chitosan as Antimicrobial Agent: Applications and Mode of Action. Biomacromolecules 2003, 4 (6), 1457–1465. 10.1021/bm034130m. 14606868

[ref54] Tiraferri A.; Maroni P.; Caro Rodríguez D.; Borkovec M. Mechanism of Chitosan Adsorption on Silica from Aqueous Solutions. Langmuir 2014, 30 (17), 4980–4988. 10.1021/la500680g. 24725003

[ref55] Kosmulski M. PH-Dependent Surface Charging and Points of Zero Charge. IV. Update and New Approach. J. Colloid Interface Sci. 2009, 337 (2), 439–448. 10.1016/j.jcis.2009.04.072. 19501839

[ref56] Lundin M.; Blomberg E.; Tilton R. D. Polymer Dynamics in Layer-by-Layer Assemblies of Chitosan and Heparin. Langmuir 2010, 26 (15), 3242–3251. 10.1021/la902968h. 19921830

[ref57] Shin P.; Sung J.; Lee M. H. Control of Droplet Formation for Low Viscosity Fluid by Double Waveforms Applied to a Piezoelectric Inkjet Nozzle. Microelectron. Reliab. 2011, 51 (4), 797–804. 10.1016/j.microrel.2010.11.017.

[ref58] Derby B. Inkjet Printing of Functional and Structural Materials: Fluid Property Requirements, Feature Stability, and Resolution. Annu. Rev. Mater. Res. 2010, 40 (1), 395–414. 10.1146/annurev-matsci-070909-104502.

[ref59] Nilsen-nygaard J.; Strand S. P.; Vårum K. M.; Draget K. I.; Nordgård C. T. Chitosan: Gels and Interfacial Properties. Polymers (Basel, Switz.) 2015, 7 (3), 552–579. 10.3390/polym7030552.

[ref60] Desbrieres J. Viscosity of Semiflexible Chitosan Solutions: Influence of Concentration, Temperature, and Role of Intermolecular Interactions. Biomacromolecules 2002, 3 (2), 342–349. 10.1021/bm010151+. 11888321

[ref61] Arrabito G.; Musumeci C.; Aiello V.; Libertino S.; Compagnini G.; Pignataro B. On the Relationship between Jetted Inks and Printed Biopatterns: Molecular-Thin Functional Microarrays of Glucose Oxidase. Langmuir 2009, 25 (11), 6312–6318. 10.1021/la900071z. 19317422

[ref62] Liu Y. F.; Tsai M. H.; Pai Y. F.; Hwang W. S. Control of Droplet Formation by Operating Waveform for Inks with Various Viscosities in Piezoelectric Inkjet Printing. Appl. Phys. A: Mater. Sci. Process. 2013, 111 (2), 509–516. 10.1007/s00339-013-7569-7.

[ref63] He B.; Yang S.; Qin Z.; Wen B.; Zhang C. The Roles of Wettability and Surface Tension in Droplet Formation during Inkjet Printing. Sci. Rep. 2017, 7, 11841 10.1038/s41598-017-12189-7. 28928447 PMC5605737

[ref64] Miccichè C.; Arrabito G.; Amato F.; Buscarino G.; Agnello S.; Pignataro B. Inkjet Printing Ag Nanoparticles for SERS Hot Spots. Anal. Methods 2018, 10 (26), 3215–3223. 10.1039/C8AY00624E.

[ref65] Ziani K.; Oses J.; Coma V.; Maté J. I. Effect of the Presence of Glycerol and Tween 20 on the Chemical and Physical Properties of Films Based on Chitosan with Different Degree of Deacetylation. Food Sci. Technol. 2008, 41 (10), 2159–2165. 10.1016/j.lwt.2007.11.023.

[ref66] Walke S.; Srivastava G.; Nikalje M.; Doshi J.; Kumar R.; Ravetkar S.; Doshi P. Physicochemical and Functional Characterization of Chitosan Prepared From Shrimp Shells and Investigation of Its Antibacterial, Antioxidant and Tetanus Toxoid Entrapment Efficiency. Int. J. Pharm. Sci. Rev. Res. 2014, 26 (38), 215–225.

[ref67] Lee H.; Eckmann D. M.; Lee D.; Hickok N. J.; Composto R. J. Symmetric PH-Dependent Swelling and Antibacterial Properties of Chitosan Brushes. Langmuir 2011, 27 (20), 12458–12465. 10.1021/la202616u. 21894981 PMC3191253

[ref68] Lavorgna M.; Piscitelli F.; Mangiacapra P.; Buonocore G. G. Study of the Combined Effect of Both Clay and Glycerol Plasticizer on the Properties of Chitosan Films. Carbohydr. Polym. 2010, 82 (2), 291–298. 10.1016/j.carbpol.2010.04.054.

[ref69] Bhattacharyya D.; Xu H.; Deshmukh R. R.; Timmons R. B.; Nguyen K. T. Surface Chemistry and Polymer Film Thickness Effects on Endothelial Cell Adhesion and Proliferation. J. Biomed. Mater. Res., Part A 2010, 94A (2), 640–648. 10.1002/jbm.a.32713. PMC2892191 20213813

[ref70] Mccloskey B. D.; Ju H.; Freeman B. D. Composite Membranes Based on a Selective Chitosan - Poly (Ethylene Glycol) Hybrid Layer: Synthesis, Characterization, and Performance in Oil - Water Purification. Ind. Eng. Chem. Res. 2010, 49, 366–373. 10.1021/ie901197u.

[ref71] Cheng Y.; Liu Y.; Huang J.; Xian Y.; Zhang Z.; Jin L. Fabrication of Tyrosinase Biosensor Based on Multiwalled Carbon Nanotubes-Chitosan Composite and Its Application to Rapid Determination of Coliforms. Electroanalysis 2008, 20, 1463–1469. 10.1002/elan.200704195.

[ref72] Na G. C. Interaction of Calf Skin Collagen with Glycerol: Linked Function Analysis. Biochemistry 1986, 25 (5), 967–973. 10.1021/bi00353a004. 3964670

[ref73] Kezwon A.; Wojciechowski K. Effect of Temperature on Surface Tension and Surface Dilational Rheology of Type I Collagen. Colloids Surf., A 2014, 460, 168–175. 10.1016/j.colsurfa.2014.05.025.

[ref74] Li Y.; Qiao C.; Shi L.; Jiang Q.; Li T. Viscosity of Collagen Solutions: Influence of Concentration, Temperature, Adsorption, and Role of Intermolecular Interactions. J. Macromol. Sci., Part B: Phys. 2014, 53 (5), 893–901. 10.1080/00222348.2013.852059.

[ref75] Eggers J. Universal Pinching of 3D Axisymmetric Free-Surface Flow. Phys. Rev. Lett. 1993, 71 (21), 3458–3460. 10.1103/PhysRevLett.71.3458. 10054982

[ref76] Arrabito G.; Cavaleri F.; Porchetta A.; Ricci F.; Vetri V.; Leone M.; Pignataro B. Printing Life-Inspired Subcellular Scale Compartments with Autonomous Molecularly Crowded Confinement. Adv. Biosyst. 2019, 3 (7), 1900023 10.1002/adbi.201900023. 32648672

[ref77] Dong H.; Carr W. W.; Morris J. F. An Experimental Study of Drop-on-Demand Drop Formation. Phys. Fluids 2006, 18 (7), 072102 10.1063/1.2217929.

[ref78] Belmiloud N.; Tamaddon A. H.; Mertens P. W.; Struyf H.; Xu X. Dynamics of the Drying Defects Left by Residual Ultra-Pure Water Droplets on Silicon Substrate. ECS J. Solid State Sci. Technol. 2012, 1, P34–P39. 10.1149/2.014201jss.

[ref79] Fischer H.; Polikarpov I.; Craievich A. F. Average Protein Density Is a Molecular-Weight-Dependent Function. Protein Sci. 2004, 13 (10), 2825–2828. 10.1110/ps.04688204. 15388866 PMC2286542

[ref80] Langenbach K. J.; Elliott J. T.; Tona A.; McDaniel D.; Plant A. L. Thin Films of Type 1 Collagen for Cell by Cell Analysis of Morphology and Tenascin-C Promoter Activity. BMC Biotechnol. 2006, 6 (1), 14 10.1186/1472-6750-6-14. 16519810 PMC1523190

[ref81] Kilian K. A.; Bugarija B.; Lahn B. T.; Mrksich M. Geometric Cues for Directing the Differentiation of Mesenchymal Stem Cells. Proc. Natl. Acad. Sci. U. S. A. 2010, 107 (11), 4872–4877. 10.1073/pnas.0903269107. 20194780 PMC2841932

[ref82] Li R.; Ashgriz N.; Chandra S. Maximum Spread of Droplet on Solid Surface: Low Reynolds and Weber Numbers. J. Fluids Eng. 2010, 132, 061302 10.1115/1.4001695.

[ref83] Kauffman M. K.; Kauffman M. E.; Zhu H.; Jia Z.; Li Y. R. Fluorescence-Based Assays for Measuring Doxorubicin in Biological Systems. React. Oxyg. species 2016, 2 (6), 432–439. 10.20455/ros.2016.873. PMC5921830 29707647

[ref84] Albrecht M. A.; Evans C. W.; Raston C. L. Green Chemistry and the Health Implications of Nanoparticles. Green Chem. 2006, 8, 417–432. 10.1039/b517131h.

[ref85] Huang T.-T.; Wu W. Scalable Nanomanufacturing of Inkjet-Printed Wearable Energy Storage Devices. J. Mater. Chem. A 2019, 7 (41), 23280–23300. 10.1039/C9TA05239A.

[ref86] Xia J.; Qiu Y.; Xun X.; Ma L.; Guan J.; Su M. Analytica Chimica Acta Single Cell Patterning for High Throughput Sub-Cellular Toxicity Assay. Anal. Chim. Acta 2018, 1007, 26–32. 10.1016/j.aca.2017.11.044. 29405985

[ref87] Zhang P.; Liu Y.; Xia J.; Wang Z.; Kirkland B.; Guan J. Top-Down Fabrication of Polyelectrolyte-Thermoplastic Hybrid Microparticles for Unidirectional Drug Delivery to Single Cells. Adv. Healthcare Mater. 2013, 2, 540–545. 10.1002/adhm.201200200. 23184769

[ref88] Meyer M. Processing of Collagen Based Biomaterials and the Resulting Materials Properties. Biomed. Eng. Online 2019, 18 (1), 24 10.1186/s12938-019-0647-0. 30885217 PMC6423854

[ref89] Patlolla A.; Collins G.; Livingston Arinzeh T. Solvent-Dependent Properties of Electrospun Fibrous Composites for Bone Tissue Regeneration. Acta Biomater. 2010, 6 (1), 90–101. 10.1016/j.actbio.2009.07.028. 19631769

