# Supplementary material for: Aqueous Processed Biopolymer Interfaces for Single-Cell Microarrays
Source: ACS Biomater Sci Eng. 2020 Apr 6;6(5):3174–86. doi: 10.1021/acsbiomaterials.9b01871 (PMC7997111; doi:10.1021/acsbiomaterials.9b01871)
Supplement: Supplementary file 1 — ab9b01871_si_001.pdf [file ab9b01871_si_001.pdf]

Supporting Information

Aqueous Processed Biopolymer Interfaces for Single-Cell Microarrays

Vittorio Ferrara,[a] Giovanni Zito,[b] Giuseppe Arrabito,[c] Sebastiano Cataldo,[c] Michelangelo Scopelliti,[c] Carla Giordano,[b] Vetri Vetri,[c] Bruno Pignataro*[c]

[a] Dipartimento di Scienze Chimiche, Università di Catania, v.le A. Doria 6, 95125 Catania, Italy.

[b] Dipartimento di Promozione della Salute, Materno-Infantile, Medicina Interna e Specialistica di Eccellenza "G. D'Alessandro" (ProMISE), Sezione di Malattie Endocrine, del Ricambio e della Nutrizione, Università di Palermo, p.za delle Cliniche 2, 90127, Palermo, Sicilia, Italy.

[c] Dipartimento di Fisica e Chimica – Emilio Segrè, Università di Palermo, V.le delle Scienze, 90128 Palermo, Italy.

Table of Contents

Biochip architecture S2

Printing condition for chitosan ink ejection S2

Derby plot for the chitosan and collagen inks S3

Fluorescence images of chitosan patterns S4

Collagen microarrays staining S4

Diameter distributions of collagen ink sessile droplets S5

Count of cells adherent on collagen array S6

References S6

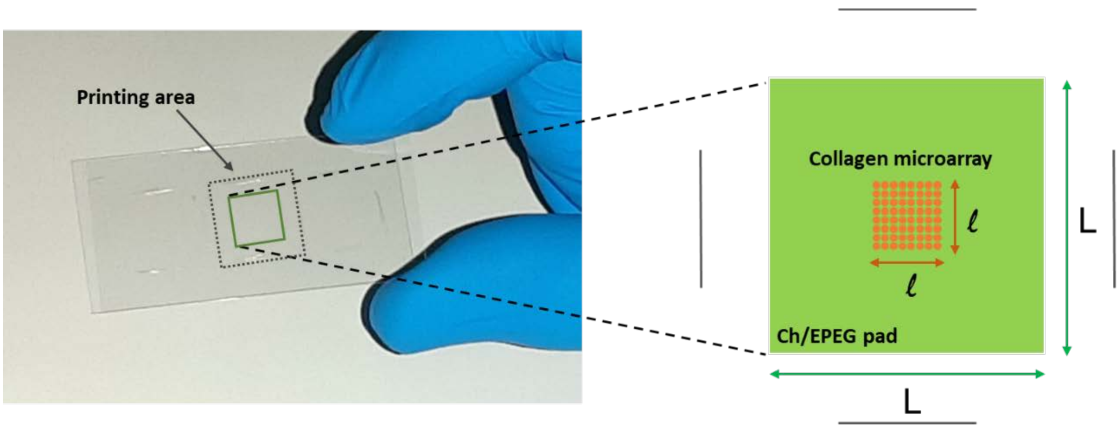

Figure S1. Biochip architecture: photograph of the biochip and scheme of the printed chitosan and collagen patterns. Typical values of L and ℓ involved in single-cell capture experiments are 8 mm and 1 mm, respectively (schematic not to scale). The collagen microarray can be printed tuning the spot size and their relative distance. As shown in the photograph, the printing area is delimited by scratches on the glass support, and multiple collagen microarrays can be obtained on a single biochip (three in the herein shown platform).

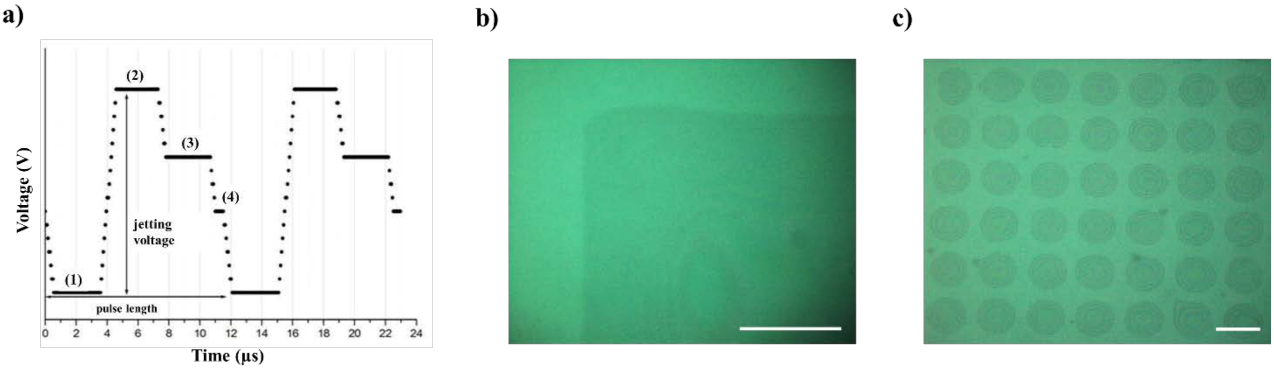

Figure S2. Printing condition for chitosan ink ejection: (a) scheme of the applied double pulse waveform, showing the four-segment voltage constituting a pulse length of 11.52 µs,[1] (b) white-field image of a portion of wet 8 x 8 mm^2 chitosan pad and (c) white-field image of wet chitosan array. Scale bars are (b) 1 mm and (c) 100 µm.

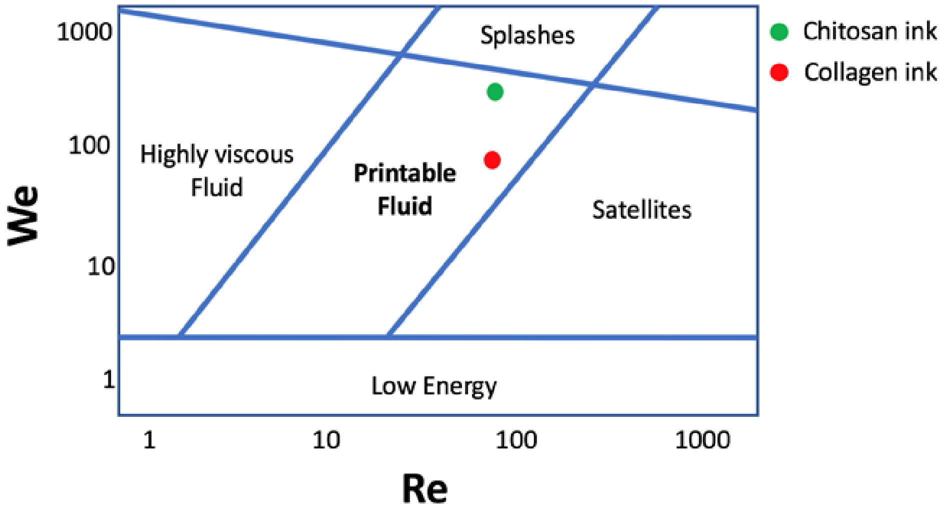

Figure S3. Derby plot for the chitosan and collagen inks. The Derby graph shows the regime of ink printability as a function of Weber ($\mathbf{We} = \frac{\rho v^2 L}{\sigma}$), and Reynolds ($\mathbf{Re} = \frac{\rho v L}{\mu}$) dimensionless numbers. The experimental points relevant to the chitosan and collagen ink droplets are marked by a green and red dot, respectively. The two dots reported are obtained by an estimation of the main parameters that describe the two experimental cases: chitosan ink (surface tension: 70 mN/m, viscosity: 10 mPa·s, density 1000 Kg/m^3, velocity of 10 pL droplet: 27.1 m/s, diameter of the droplet: 22 μm) and collagen ink (surface tension: 70 mN/m, viscosity: 3 mPa·s, density 1000 Kg/m^3, velocity of 10 pL droplet: 6 m/s, diameter of the droplet: 8 μm, velocity of the 1 pL droplet: 20.0 m/s, diameter of the 1 pL droplet: 12 μm). In both cases, the dots lie within the printable area of the Derby plot, demonstrating that both the inks have good printability properties.

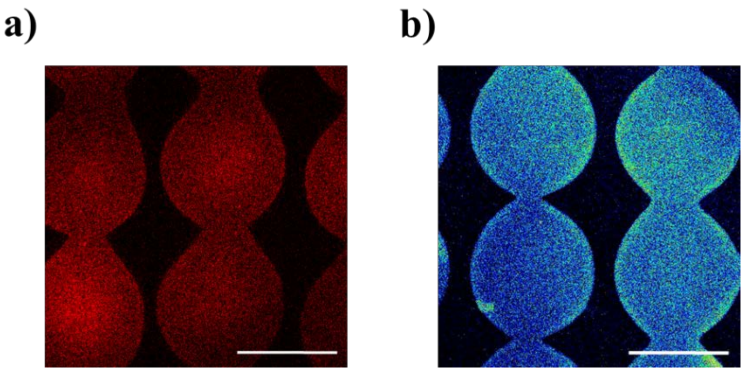

Figure S4. Fluorescence images of chitosan patterns: 15 min of incubation with standard dyes such as (a) Alexa 647, and (b) FITC-BSA, both 2 μM. The array geometry is suitable for an easier visualization of deposited chitosan by fluorescence. Chitosan swelling[2] in solution determined the spots contacted each other. Scale bars 100 μm.

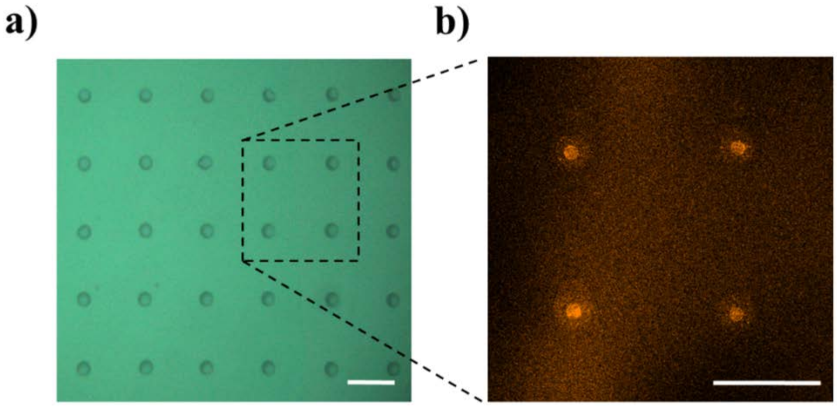

Figure S5. Collagen microarrays staining: (a) white-field image of collagen ink droplets printed by the short-pulse waveform, and fluorescence image of dried arrays stained with Sypro Orange. The inkjet printed arrays shown are characterized by spots of ~ 32 μm with 150 μm as drop spacing. The fluorescent background is due to the interaction of the dyes with the chitosan which constitutes the underlying layer. Scale bars are both 100 μm.

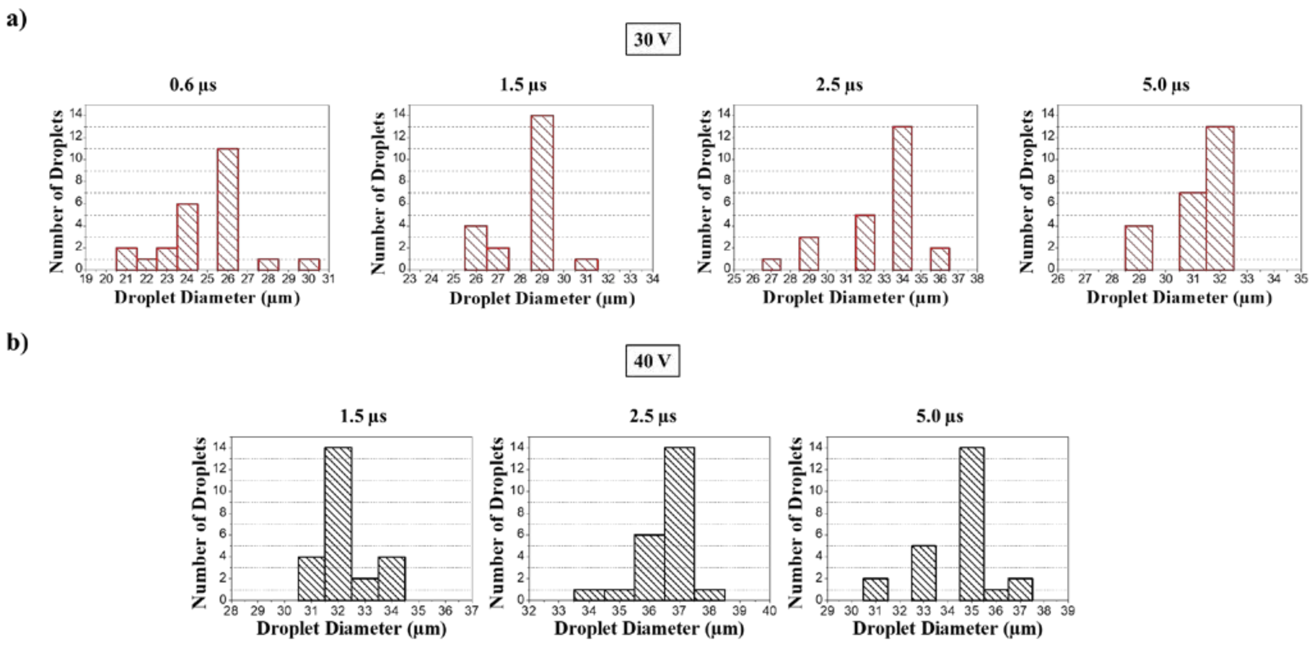

Figure S6. Diameter distributions of collagen ink sessile droplets: diameter of collagen (0.08 % w/v) ink sessile droplets on the chitosan-g-EPEG coating, printed at (a) 30 V, and (b) 40 V, by varying the t_D to tune the spot dimension (corresponding patterns in Fig. 5b and 5c).

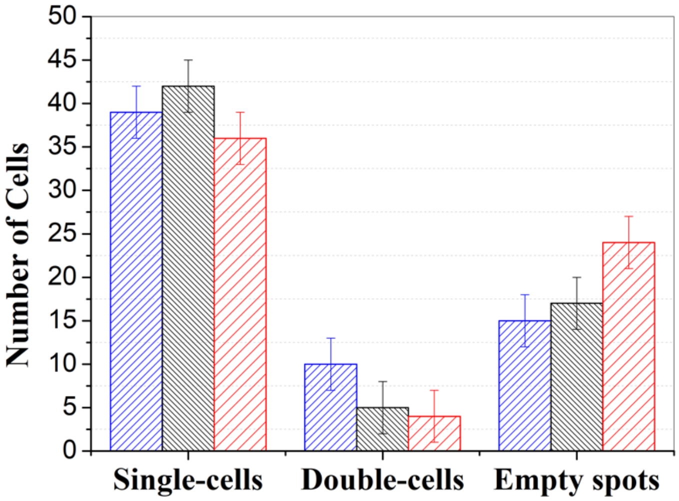

Figure S7. Count of cells adherent on collagen array: single-cell yield on 8 x 8 spots collagen microarray (spots diameter 32 µm), and biological evolution up to 48 hours after the adhesion. Data are reported in terms of number of cells, distinguishing in single-cells, double-cells and empty spots, counted at 1 h (blue), 24 h (black) and 48 h (red) after the adhesion. The number of single-cells after 1 h was 39, and reached 42 single-cells after 24 hours, likely because of the spreading of one cell on a double occupied spot, process which induced detaching of the second cell from the spot. After 48 hours a slight general loss of cell from the platform was observed, reducing the single-cell percentage at 36. Error bars indicate the standard deviation related to the mean number of counted cells.

References

1. G. Arrabito, F. Cavaleri, V. Montalbano, V. Vetri, M. Leone, and B. Pignataro, *Lab Chip*, 2016, **16**, 4666–4676.

2. X. Qu, A. Wirsén, and A.-C. Albertsson, *Polymer*, 2000, **41**, 4589–4598.
